# Imprinting at the *KBTBD6* locus involves species-specific maternal methylation and monoallelic expression in livestock animals

**DOI:** 10.1186/s40104-023-00931-3

**Published:** 2023-10-11

**Authors:** Jinsoo Ahn, In-Sul Hwang, Mi-Ryung Park, Seongsoo Hwang, Kichoon Lee

**Affiliations:** 1 Department of Animal Sciences, The Ohio State University, Columbus, OH 43210 USA; 2 Animal Biotechnology Division, Rural Development Administration, National Institute of Animal Science, Jeonbuk, 55365 Republic of Korea; 3 Columbia Center for Translational Immunology, Columbia University Irving Medical Center, Columbia University, New York, NY 10032 USA; 4 Animal Welfare Research Team, National Institute of Animal Science, RDA, 1500, Kongjwipatjwi-ro, Jeollabuk-do, 55365 Republic of Korea

**Keywords:** Differentially methylated region, Domesticated mammal, Imprinting, *KBTBD6*, Parthenogenetic

## Abstract

**Background:**

The primary differentially methylated regions (DMRs) which are maternally hypermethylated serve as imprinting control regions (ICRs) that drive monoallelic gene expression, and these ICRs have been investigated due to their implications in mammalian development. Although a subset of genes has been identified as imprinted, in-depth comparative approach needs to be developed for identification of species-specific imprinted genes. Here, we examined DNA methylation status and allelic expression at the *KBTBD6* locus across species and tissues and explored potential mechanisms of imprinting.

**Results:**

Using whole-genome bisulfite sequencing and RNA-sequencing on parthenogenetic and normal porcine embryos, we identified a maternally hypermethylated DMR between the embryos at the *KBTBD6* promoter CpG island and paternal monoallelic expression of *KBTBD6*. Also, in analyzed domesticated mammals but not in humans, non-human primates and mice, the *KBTBD6* promoter CpG islands were methylated in oocytes and/or allelically methylated in tissues, and monoallelic *KBTBD6* expression was observed, indicating livestock-specific imprinting. Further analysis revealed that these CpG islands were embedded within transcripts in porcine and bovine oocytes which coexisted with an active transcription mark and DNA methylation, implying the presence of transcription-dependent imprinting.

**Conclusions:**

In this study, our comparative approach revealed an imprinted expression of the *KBTBD6* gene in domesticated mammals, but not in humans, non-human primates, and mice which implicates species-specific evolution of genomic imprinting.

**Supplementary Information:**

The online version contains supplementary material available at 10.1186/s40104-023-00931-3.

## Background

Genomic imprinting plays a crucial role in the normal development and growth of mammals [1]. The epigenetic mechanisms underlying genomic imprinting include DNA methylation during mammalian embryonic development [2, 3]. Differentially established DNA methylation in the two parental germlines that survives demethylation during pre-implantation stages consists of epigenetic imprints and forces monoallelic expression of genes in close proximity [4]. As such, the primary differentially methylated regions (DMRs) established in the germlines are often maternally hypermethylated (e.g., the DMR at the *SGCE*/*PEG10* locus serves as the imprinting control region (ICR) that drives paternal expression of these two genes), and these ICRs have been investigated due to their implications in mammalian growth and development [5–10]. However, although imprinted genes have been identified first in mice and humans in most cases and a subset of orthologous loci is imprinted in domesticated animals [11], there can be livestock-specific imprinting that has not been investigated. Our recent approach of the use of whole-genome bisulfite sequencing (WGBS) and RNA sequencing (RNA-seq) of porcine embryos that underwent parthenogenesis has facilitated verification of imprinting status of gene clusters [9, 12]. This facilitation was attributed to a direct sequencing comparison between parthenogenetic and biparental porcine embryos.

The Kelch superfamily of proteins including the Kelch repeat and BTB domain-containing protein (KBTBD) are involved in a number of cellular processes such as cell development, cytoskeletal arrangement, and protein degradation [13]. Among the Kelch family members, KBTBD6 is one of the adapters that is required for assembly of the CUL3-KBTBD6/KBTBD7 ubiquitin ligase complex, which ubiquitylates T-lymphoma and metastasis gene 1 (TIAM1) and targets the protein to proteasomal degradation [14]. Subsequently, the degradation of TIAM1, a guanine exchange factor (GEF) specific for RAC1 activation, leads to inactivation of RAC1-mediated signaling that controls a variety of cellular responses including actin rearrangements, cell motility, and cell proliferation [15, 16]. Detailed imprinting status of *KBTBD6* in mammalian species including humans and mice has yet to be investigated, and recently, paternally preferential expression of *KBTBD6* in pigs [17] and partial allelic methylation of the *KBTBD6* promoter CpG island in one tissue (dermal fibroblast) from dogs, cows, and pigs [18] have been documented. However, whether the paternal expression of *KBTBD6* in pigs occurs concurrently with maternal imprints, i.e., maternal methylation, in the promoter region remains to be examined. In addition, whether methylation in oocytes, but not in sperm, occurs in the *KBTBD6* loci and what could be the mechanism of de novo DNA methylation in the maternal allele of the *KBTBD6* promoter have not been investigated.

Here, we aimed to determine differential DNA methylation patterns within the potential promoter region of *KBTBD6* between parthenogenetic and normal biparental pig embryos using WGBS. We found that a maternally hypermethylated DMR encompasses the *KBTBD6* promoter in pigs and the porcine *KBTBD6* expression is paternal allele-specific. Noticeably, within the *KBTBD6* locus, maternal methylation in oocytes and/or allelic methylation in tissues was only found in analyzed domesticated mammals, but not in humans, non-human primates, and mice. Subsequently, monoallelic or biallelic expression of the *KBTBD6* gene and neighboring genes was investigated by analyzing genome sequencing and RNA-seq data from the same individuals across mammalian species. Furthermore, the potential mechanisms of de novo DNA methylation in porcine and bovine oocytes were investigated in relation to active transcription at the *KBTBD6* promoter. Our results highlight imprinting in the *KBTBD6* locus which may operate in domesticated mammals via paternal monoallelic expression.

## Methods

### Ethics statement

All animal procedures were approved by the Institutional Animal Care and Use Committee (IACUC) of the National Institute of Animal Science, Rural Development Administration (RDA) of Korea (NIAS2015-670).

### Sample collection

The method of in vitro maturation (IVM) of pig oocytes and production of parthenogenetic embryos has been described in our previous reports [19, 20]. In brief, ovaries from Landrace × Yorkshire × Duroc (LYD) pigs were obtained from a local slaughterhouse, and cumulus-oocyte complexes (COCs) were collected and washed in Tyrode's lactate-Hepes containing 0.1% (w/v) polyvinyl alcohol. For IVM, 50 COCs washed three times in TCM-199 based medium (GIBCO, Grand Island, NY, USA) were placed in each well of four-well dishes (Nunc, Roskilde, Denmark) containing 500 µL of maturation medium and matured for 40–42 h at 38.5 °C in an incubator containing 5% CO_2_. Then, cumulus cells were removed, and oocytes having the first polar body were selected and placed in a fusion chamber with 250 µm diameter wire electrodes (BLS, Budapest, Hungary) covered with 0.3 mol/L mannitol solution. The fusion was achieved by two DC pulses (1 s interval) of 1.2 kV/cm for 30 µs using an LF101 Electro Cell Fusion Generator (Nepa Gene Co., Ltd., Chiba, Japan). After 2 h of stabilization, 200 parthenogenetic (PA) embryos were placed into oviducts of each of the two LYD surrogate gilts aged 12 months at the onset of estrus to develop the embryos. To produce fertilized control (CN) embryos, two LYD gilts were naturally mated with boars twice with a 6-h interval during the natural heat period at the onset of estrus. The PA and CN embryos were recovered from the euthanized surrogates and gilts, respectively, at d 21 from the onset of estrus to ensure occurrence of monoallelic expression after the blastocyst stage [21] and non-occurrence morphological changes of parthenogenetic embryos which are shown at approximately d 30 [22]. The recovery was carried out by sectioning their reproductive tracts, isolating placenta from the uterus, and separating embryos from the surrounding placenta. Morphologically intact embryos with comparable sizes were selected for further experiments and stored in liquid nitrogen until further use.

### Whole-genome bisulfite sequencing

DNA methylomes were constructed independently for each individual in each CN and PA group to reduce genetic variability. For WGBS data generation, genomic DNA was isolated from the whole collected CN and PA embryos (two biological replicates for each group) and fragmented. The fragmented DNA (200 ng) was subjected to bisulfite conversion using the EZ DNA Methylation-Gold Kit (Zymo Research, Irvine, CA, USA). The Accel-NGS Methyl-Seq DNA Library Kit (Swift Biosciences, Inc., Ann Arbor, MI, USA) was used to construct the DNA library using 1 ng of DNA according to the manufacturer's instructions. PCR was conducted with adapter primers and Diastar™ EF-Taq DNA polymerase (Solgent, Daejeon, Korea) under the following thermal conditions: 3 min at 95 °C followed by 10 cycles of 30 s at 95 °C, 30 s at 60 °C, and 30 s at 72 °C, and a final extension for 5 min at 72 °C. After bead-based clean-up, the DNA library was sequenced to generate 151-nucleotide paired-end reads using HiSeqX sequencer operated by Macrogen Inc. (Seoul, Korea). The raw reads were trimmed and filtered out to remove adapters and reads shorter than 20 bp by using Trim Galore (v0.4.5), remaining 846.5 (CN1), 866.5 (CN2), 839.7 (PA1), and 849.2 (PA2) million cleaned reads. Mapping to the pig reference genome (susScr11) was conducted using the Bismark aligner (v0.22.3) [23] with default parameters and, after deduplication of 14.30%, 14.58%, 14.82%, and 13.00% of alignments for CN1, CN2, PA1, and PA2 embryos, respectively, using deduplicate_bismark, the methylation ratio of every cytosine in CpGs was extracted using the Bismark methylation extractor with default settings including ‘–no_overlap’ for paired-end reads.

### RNA sequencing

To produce transcriptome, RNA-seq was performed with total RNA isolated from the whole collected CN and PA embryos (two biological replicates for each group) using TRIzol reagent (Sigma-Aldrich, USA) following the manufacturer's instructions. The RNA samples, treated with Dnase I to avoid genomic DNA contamination, were electrophoresed in 1.2% agarose gels to evaluate the integrity of RNA, which was then confirmed by more than two of 28S/18S rRNA ratio and more than 7 of RNA integrity number (RIN) using an Agilent 2100 BioAnalyzer. Using the ratios of A_260/280_ and A_260/230_ (1.8–2.0), the concentrations of RNA were assessed. One μg of total RNA was used to construct cDNA libraries with the TruSeq RNA Sample Prep Kit v.2 (Illumina, San Diego, CA, USA). Quantification of the cDNA libraries was done by quantitative Real-Time PCR (qPCR) according to the qPCR Quantification Protocol Guide, and qualification of the libraries was assessed using the Agilent 2100 Bioanalyzer. The Illumina HiSeq2500 RNA-seq platform was used to sequence the library products (100 nt paired-end). After adapter trimming and quality filtering, the number of remaining cleaned reads were approximately 77.2 (CN1), 73.3 (CN2), 80.5 (PA1), and 80.7 (PA2) million cleaned reads were retrieved. STAR aligner (v2.7.5) [24] was used to align the reads to the reference genome (susScr11) with default parameter settings.

### Analysis of WGBS and RNA-seq

For WGBS analysis, the program metilene (v0.2–8) [25] was used to identify methylated regions (MRs) passing criteria of a genomic distance of less than 300 bp between CpGs, a presence of more than 10 CpGs, and a mean methylation difference of more than 0.2 between CN and PA groups. Among MRs, differentially methylated regions (DMRs) satisfied false discovery rate (FDR) < 0.05. Methylation ratios from WGBS were depicted using the R/Bioconductor package Gviz (v1.28.3) [26]. For analysis of RNA-seq, BAM files of aligned reads were produced following deduplication using Picard MarkDuplicates and quality-filtering using SAMtools [27] (-q 30). BAM file-derived read coverages from RNA-seq were normalized to transcripts per million (TPM) using bamCoverage in deepTools with parameters (–binSize 10, –smoothLength 15) [28] and then visualized using Gviz [26]. Transcript quantification was performed using Salmon (v1.3.0) with the mapping-based mode [29]. TPM values of each gene were obtained from Salmon output files (quant.sf).

### Data mining and processing

Publicly available data were downloaded from the NCBI GEO unless otherwise stated. For the human, data for oocytes and sperm were downloaded under accession number GSE85632 (RNA-seq) [30], GSE124718 (H3K4me3) [31], and GSE81233 (WGBS) [32]. WGBS data from human somatic tissues were derived from GSE17312 [33]. For rhesus monkeys, data under GSE112536 (oocyte RNA-seq) [34], GSE60166 (oocyte and sperm WGBS) [35], GSE77124 (brain WGBS) [36], and GSE115065 (blood WGBS) [37] were downloaded. For the mouse, data for oocytes and sperm were downloaded: GSE71434 (RNA-seq and H3K4me3) [38], GSE112622 (H3K36me3) [39], GSE185579 (WGBS for C57BL/6 J) [40] and DRA006680 from DNA Databank of Japan (DDBJ) (WGBS for CAST/EiJ) [39]. WGBS data from mouse somatic tissues were derived from the Mouse ENCODE Project under accession no. GSE188027 (liver), GSE187995 (brain), GSE188220 (kidney), GSE188068 (heart), and GSE187979 (lung) [41]. For pigs, data for oocytes, sperm and embryos were downloaded: CRA004237 from Genome Sequence Archive (GSA) (RNA-seq of pig oocytes [42]), GSE163620 (H3K4me3, H3K36me2, and H3K36me3 of pig oocytes) [43] GSE163709 (H3K4me3 of pig 4-cell, 8-cell, and blastocyst embryos) [42], and GSE143850 (WGBS) [44]. WGBS data for pig somatic tissues were obtained from GSE157045 (skeletal muscle) [45] and PRJEB42772 (fetal and neonatal brain). For cows, oocyte, sperm, and embryo data were downloaded: GSE163620 (RNA-seq and ChIP-seq) [43] and GSE143850 (WGBS) [44]. WGBS data for cow somatic tissues were derived from GSE180592. For other species, WGBS data from tissues were analyzed: crab-eating macaque (GSE159347) [46], chimpanzee (GSE151768 and GSE112356) [47, 48], gibbon (GSE115065) [37], horse (GSE63330) [49], dog (GSE63330) [49], sheep (PRJNA622418) [50], and goat (SRR5574289 and SRR5574293 from NCBI SRA) [51]. For rat oocytes, raw RNA-seq data under GSE163620 [43] were processed.

WGS (or Exome-seq) and RNA-seq from the same individuals were analyzed: human normal lung (PRJNA395106), human normal liver (hum0158.v2 from the NBDC Human Database) [52], rhesus monkey tissues (GSE34426, GSE42857, and SRP039366) [53], rhesus monkey LCL (PRJNA563344) [54], and chimpanzee LCL (PRJNA563344) [54], dog tissues (PRJNA396033) [55], pig tissues (PRJNA493166) [56], and cow tissues (ERP118133, GSE62160, and GSE62159) [57, 58]. Mouse WGS or Exome-seq were derived from PRJNA705216 (C57BL/6 J) [59], ERP000042 (CAST/EiJ) [60], and PRJNA323493 (PWK/PhJ and CZECHII/EiJ) [61], and RNA-seq of testis from offspring of crosses of CAST/EiJ and C57BL/6 J (SRP020526) [62] and PWK/PhJ and CZECHII/EiJ (PRJNA286765) [63] were analyzed. These datasets are also listed in Additional file 1: Supplementary Table 1.

Reference genomes used in this study were hg38 (human), macFas5 (crab-eating macaque), panTro6 (chimpanzee), rheMac8 (rhesus monkey), Asia_NLE_v1 (gibbon), mm39 (mouse), equCab3 (horse), canFam3 (dog), susScr11 (pig), bosTau9 (cow), oriAri4 (sheep) and ARS1 (goat). WGBS and RNA-seq data were processed as above. ChIP-seq data were processed as described in our previous report [64] and read coverages in BAM files were normalized to 1 × depth (reads per genomic content, RPGC) using bamCoverage in deepTools with parameters (–binSize 10, –smoothLength 15, –extendReads 150) [28]. Peaks were visualized using Gviz [26]. Sequencing reads were aligned to these reference genomes or the UCSC liftOver tool was used to convert data aligned to previous genomes to those reference genomes. Information about reported SNPs was obtained as VCF files from the NCBI FTP site (human, https://ftp.ncbi.nih.gov/snp/.redesign/latest_release/VCF; other species, https://ftp.ncbi.nlm.nih.gov/snp/organisms/archive), and reported mouse SNPs from strains were derived from the Mouse Genome Informatics resource (http://www.informatics.jax.org/snp) and the EBI FTP site (http://ftp.ebi.ac.uk/pub/databases/eva/rs_releases/release_4/by_species/mus_musculus/GRCm39) [65]. VCF files aligned to previous genomes were converted to the above reference genomes using the Picard LiftoverVcf (v2.23.8). The LTR retrotransposon data were retrieved from the UCSC genome browser database [66].

### Analysis of allelic DNA methylation

Read-based analysis on partially methylated domains (PMDs) was performed to identify allelic DNA methylation as described previously [46, 67]. At first, PMDs encompassing CpG islands in promoter regions of *KBTBD6* were determined using methylation ratios (ranging from 0.3 to 0.7) from WGBS. Methylation levels of each read overlapping PMDs were calculated using MethylDackel [68]. Reads with at least 3 CpGs were qualified, and PMDs with more than 30 qualified reads were further analyzed. The number of qualified reads with methylation levels ranging either from 0 to 0.2 (hypomethylated reads) or 0.8 to 1.0 (hypermethylated reads) was divided by the total number of qualified reads to obtain percentages. PMDs with percentages more than 30 for both hypomethylated and hypermethylated reads were identified as allelically methylated regions (Additional file 2: Supplementary Table 2) [46]. For specific consecutive CpG sites within the allelically methylated regions, lollipop plots were drawn using the QUMA quantification tool for methylation analysis [69].

### Phylogenetic analysis

The phylogenetic trees with estimated divergence time were generated by TimeTree 5 and Newick files were downloaded (http://www.timetree.org, accessed on 20 July 2022) [70]. These phylogenetic trees were edited using FigTree (v.1.4.4) [71].

### Statistical analysis

For differential gene expression analysis, the Salmon output files were imported and analyzed using the R/Bioconductor package DESeq2 (v.1.28.1) [72]. Differentially expressed genes (DEGs) were obtained under the combined criteria of the absolute log_2_(fold change) > 1 and FDR < 0.05 which was regarded as a statistical significance.

## Results

### Profiling DNA methylation and gene expression led to detection of DMRs and imprinted expression

**Fig. 1:**
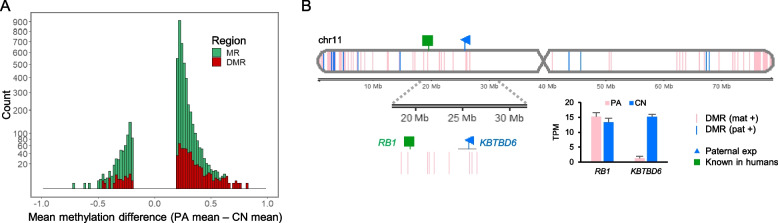
Overview of porcine methylome and transcriptome studies. **A** A histogram of mean methylation difference between PA and CN embryos in chromosome 11 (chr11) plotted against count (square root transformed y-axis) of methylated regions (MRs; satisfying distance between CpGs < 300 bp, > 10 CpGs, and mean methylation difference > 0.2, as defined in Methods). Differentially methylated regions (DMRs; FDR < 0.05) among MRs are overlaid. **B** DMRs between PA and CN embryos and expression patterns identified in chr11. Gene expression levels from RNA-seq is presented in transcripts per million (TPM). DMR (mat +), maternally hypermethylated DMR; DMR (pat +), paternally hypermethylated DMR; exp, expression; Known, known imprinted gene

After conducting parthenogenesis and normal fertilization, we obtained single-base resolution methylome by WGBS to detect DMRs (Additional file 3: Supplementary Table 3). Among methylated regions (MRs) in porcine chromosome 11, more hypermethylated DMRs (FDR < 0.05) in parthenogenetically activated (PA) embryos, an indicative of maternal methylation, were identified than hypermethylated DMRs in control (CN) embryos, an indicative of paternal methylation (Fig. 1A and Additional file 4: Supplementary Fig. 1). On the other hand, less hypermethylation in CN embryos indicated the presence of less paternal methylation from the paternal allele only existed in the CN embryo. These maternal and paternal DMRs were aligned in a chromosomal context with nearby maternal or paternal expression detected by comparison of RNA-seq of those PA and CN embryos (Fig. 1B and Additional file 3: Supplementary Table 3). Our stringent matches of maternal methylation to paternal expression or paternal methylation to maternal expression for detecting direct imprinting effects on gene expression, through searching for a DMR located within 2 kb upstream of the transcription start site (TSS) and 1 kb downstream of the TSS (a 3 kb-window), led to identification of genomic imprinting at the *KBTBD6* locus (Fig. 1B). This locus is located around the *RB1* locus which is known to be imprinted in humans [73, 74]. Taken together, our generation of PA and CN embryos resulted in an efficient comparison of methylation of parental alleles and identification of DMRs and imprinted expression.

### A DMR exists near the porcine *KBTBD6* gene but not at the orthologous human locus, which oppositely occurred in relation to the *RB1* locus

**Fig. 2:**
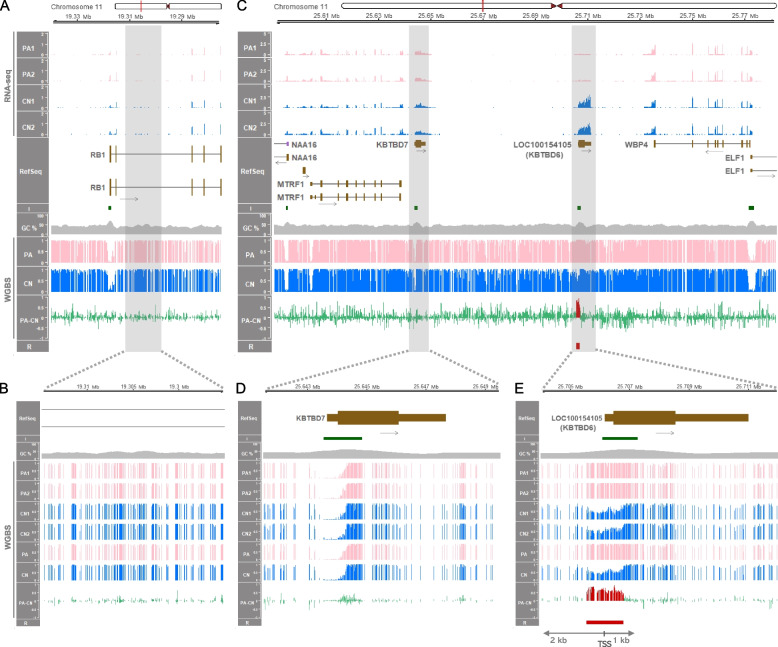
Gene expression and DNA methylation along the porcine *RB1* and *KBTBD6* loci. **A** The 65-kb region containing the *RB1* locus between 19.275 Mb (19,275,000) and 19.34 Mb (19,340,000) based on NCBI RefSeq annotation. RNA-seq read coverages of the *RB1* transcripts in PA and CN embryos are presented in values of TPM. Mean methylation ratios based on WGBS are followed by mean methylation differences (PA-CN). **B** A close view of the 2^nd^ intron area of the *RB1* gene and methylation status. **C** The 192-kb region containing the *KBTBD6* locus between 25.59 Mb (25,590,000) and 25.782 Mb (25,782,000). RNA-seq read coverages of *KBTBD6* and surrounding transcripts in TPM, and mean methylation ratios based on WGBS. **D** and **E** Zoomed-in views of promoter regions of the *KBTBD7* and *KBTBD6* genes. R, DMR (FDR < 0.05) in red. Also, the DMR (hypermethylated in PA) is overlaid with red histogram lines in PA-CN. Red vertical bars in the ideogram, chromosomal locations; I, CpG island; GC%, GC content; black arrows, transcriptional direction; brown boxes, protein-coding transcripts (tall, translated region; short, untranslated region); purple boxes, noncoding transcripts; PA1–2, individual PA embryos; CN1–2, individual CN embryos; PA, mean methylation ratio for PA embryos; CN, mean methylation ratio for CN embryos

As the *RB1* locus is located relatively closely to the *KBTBD6* locus and detailed imprinting status of both loci has not been compared between humans and pigs, the *RB1* and *KBTBD6* loci were examined closely by analyzing WGBS data. In humans, *RB1* is expressed preferentially from the maternal allele where the CpG island in intron 2 is methylated and silenced, while the alternative transcript 2B is expressed from the unmethylated CpG island in intron 2 in the paternal allele. This maternal *RB1* expression is potentially due to transcriptional interference on the paternal allele via binding of transcription complex to the unmethylated paternal allele as a roadblock for the full-length *RB1* transcript [73, 75]. In the 2^nd^ intron region of the human *RB1* gene in oocytes, there was a maternally methylated CpG island in intron 2 as a part of the *PPP1R26P1* element (the retrocopy of the *PPP1R26* gene) which was integrated in reverse orientation relative to *RB1* by a retrotransposition event (Additional file 4: Supplementary Fig. 2). The *PPP1R26* gene also exists in the pig and is located in the unplaced scaffold (NW_018084833.1) of the current pig genome assembly (susScr11) (Additional file 4: Supplementary Fig. 3). However, in pigs, the orthologous *RB1* intron 2 did not contain the retrotransposed *PPP1R26P1* element and was not differentially methylated (Fig. 2A and B and Additional file 4: Supplementary Fig. 4A). In summary, there was no conserved intronic element that can affect expression of the pig *RB1* gene. Along the *KBTBD6* locus, the order of protein-coding genes in the human chromosome 13 (13q14.11) and mouse chromosome 14 (14 D3) (i.e., *MTRF1*, *KBTBD7*, *KBTBD6*, and *WBP4*) is conserved in the pig chromosome 11. Based on the genome sequence of *Sus scrofa* (Sscrofa11.1), those four protein-coding genes are mapped to an approximate 170-kb region between approx. 25.60 Mb and 25.77 Mb (25,603,784–25,772,555) and *KBTBD7* and *KBTBD6* (aka. *LOC100154105*) are intronless (Fig. 2C). There are three CpG islands in this locus within putative promoter regions of *KBTBD7*, *KBTBD6*, and *WBP4* (and *ELF1*), and only the area containing a putative promoter region of *KBTBD6* was differentially methylated between PA and CN embryos (Fig. 2C). A close view of a putative promoter region of *KBTBD7* encompassing TSS displayed that the area with a CpG island and high GC content was regionally hypomethylated or almost unmethylated in both PA and CN embryos (Fig. 2D). It indicated that the promoter of *KBTBD7* is biallelically active and the porcine *KBTBD7* gene is not imprinted. To the contrary, the putative promoter region of *KBTBD6* was hypermethylated in PA embryos within the 3 kb-window (Fig. 2E). Considering that PA embryos have two maternal alleles and CN embryos contain one paternal and one maternal allele, the hypermethylation in PA embryos might originate from methylation on the two maternal alleles. In addition, methylation on CN embryos which was in an almost half degree might also be derived from methylation on the one maternal allele. As a result, a DMR was identified in the putative promoter region of *KBTBD6* and it might be methylated only in the maternal allele, but not in the paternal allele, suggesting that this region is being maternally inactivated by the maternal imprint.

In addition, putative promoter regions of the *WBP4* and *MTRF1* genes showed hypomethylation or unmethylation in both PA and CN embryos, suggesting biallelically activated status of these promoters and the non-imprinting of these genes (Fig. 2C). On the other hand, in humans, there are four CpG islands within the orthologous locus and all of them were hypomethylated or almost unmethylated in oocytes, sperm, blastocyst, fetal tissues (fetal brain and fetal muscle), and adult tissues (brain, muscle, heart, liver, and lung) (Additional file 4: Supplementary Fig. 5). Taken together, it indicated that the maternally methylated DMR at the *KBTBD6* promoter CpG island is porcine-specific and not conserved in humans, as well as locus-specific in the pig chromosome 11.

### Within the analyzed loci, expression of the *KBTBD6* gene only in bi-parental embryos, but not in uni-maternal embryos, indicates paternal allele expression

To examine changes in gene expression in uni-maternal PA embryos compared to bi-parental CN embryos, RNA-seq was conducted. Without imprinting, the porcine *RB1* gene was expressed in both PA and CN embryos indicating expressions from both alleles (Fig. 2A) and biallelically expressed in analyzed tissues (Additional file 4: Supplementary Fig. 4B). It implicated that the dosage of the retinoblastoma tumor-suppressor gene, *RB1* [76], is not epigenetically regulated in pigs. The *KBTBD6* gene was exclusively expressed in CN embryos, but not in PA embryos (Fig. 2C). Given that the putative promoter region of *KBTBD6* was apparently maternally inactivated by the maternal imprint (maternal methylation), the expression of the *KBTBD6* gene at a very low level in PA embryos might be due to inhibition of gene expression in the two maternal alleles. In contrast, the exclusive expression of the *KBTBD6* gene in CN embryos might be attributed to paternal allele-specific expression while the expression from the maternal allele is absent or very low. Other protein-coding genes within the porcine *MTRF1*-*WBP4* region (*MTRF1*, *KBTBD7*, and *WBP4*) were expressed in both PA and CN embryos and the expression levels were comparable between PA and CN embryos (Fig. 2C), indicating that the expression of these genes occurs in both the paternal and maternal alleles at similar degrees (biallelic expression). To quantify the expression degrees, differential expression of the genes was analyzed. Among the four protein-coding genes in the *MTRF1*-*WBP4* region, the *KBTBD6* gene was a differentially expressed gene (DEG) between PA and CN embryos (FDR < 0.001) and the expression in CN embryos was 11.8-fold higher than in PA embryos (Additional file 4: Supplementary Fig. 6). In contrast, expression levels of other genes were not statistically different between PA and CN embryos (Additional file 4: Supplementary Fig. 6), indicating that imprinted expression that might be related to the aforementioned DMR occurred solely in the *KBTBD6* gene. Moreover, positive controls of known maternal imprinting (*SGCE* and *PEG10*) showed exclusive expression in CN embryos (paternal expression) and maternal DNA methylation encompassing the promoter regions (Additional file 4: Supplementary Fig. 7A). To the contrary, a negative control (a *GNAS* isoform, also known as *NESP*) showed approximately 1.5-fold greater expression in PA embryos on average and DNA methylation exclusively in CN embryos (paternal DNA methylation) along with methylation canyon in PA embryos (Additional file 4: Supplementary Fig. 7B). While the expression of *NESP* was expected to be twofold greater in PA embryos, the varying detected level of maternal expression in parthenogenetic ovine fetuses compared to controls (from 1.7-fold to 4.3-fold) was also previously reported [77] as in our case. These controls further support our findings of imprinting at the *KBTBD6* locus.

### Mammalian DNA methylation at the *KBTBD6* locus shows oocyte- and species-specificity

**Fig. 3:**
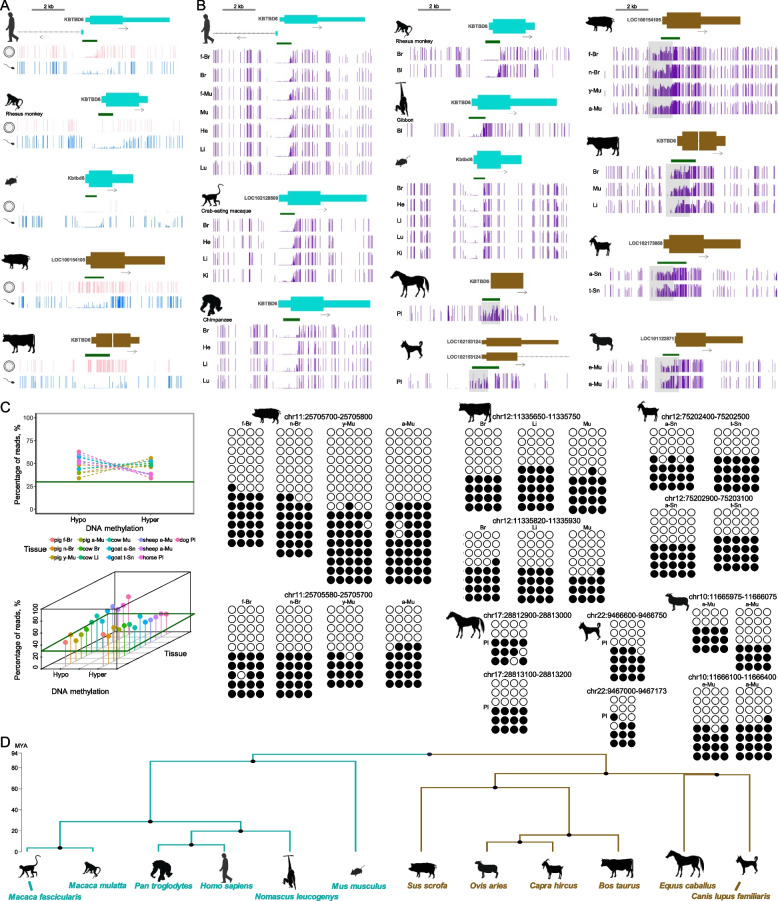
Comparison of single-base resolution DNA methylomes at the *KBTBD6* locus among mammalian species. **A** DNA methylation in oocytes (pink histogram lines) and sperm (blue histogram lines) of the human (*Homo sapiens*), rhesus monkey (*Macaca mulatta*), mouse (*Mus musculus*), pig (*Sus scrofa*), and cow (*Bos taurus*) are displayed encompassing the *KBTBD6* promoter CpG islands. **B** DNA methylation in fetal, neonatal and/or adult tissues. Species other than in **A** include crab-eating macaque (*Macaca fascicularis*), chimpanzee (*Pan troglodytes*), gibbon (*Nomascus leucogenys*), horse (*Equus caballus*), dog (*Canis lupus familiaris*), goat (*Capra hircus*), and sheep (*Ovis aries*). Analyzed tissues include: f-Br, fetal brain; Br, brain; f-Mu, fetal muscle; Mu, muscle; He, heart; Li, liver; Lu, lung; Ki, kidney; Bl, blood; Pl, placenta; n-Br, neonatal brain; y-Mu, young skeletal muscle (d 40); a-Mu, adult skeletal muscle (d 180); a-Sn, angen stage of skin; and t-Sn, telogen stage of skin; e-Mu, embryonic muscle (embryonic d 110); a-Mu (sheep), adult skeletal muscle (2-year-old). **C** Read-based analysis on partially methylated domains (PMDs) [pig chr11:25,705,500–25706700, cow chr12:11,335,500–11,336,700, goat chr12:75,202,000–75,203,500, sheep chr10:11,665,250–11,666,500, horse chr17:28,812,781–28,813,430, and dog chr22:9,466,000–9,467,173; These regions are grey-shaded in **B**]. The number of qualified reads (at least 3 CpGs) with methylation levels ranging either from 0.0 to 0.2 (hypomethylated reads) or 0.8 to 1.0 (hypermethylated reads) was divided by the total number of qualified reads to plot percentages of hypo- and hyper-methylated reads in PMDs in each tissue. A threshold for allelically methylated regions was the percentage of 30%. Consecutive CpG sites (*x*-axis) within the allelically methylated regions are plotted for each read (*y*-axis) with open and closed circles indicating unmethylated and methylated CpG sites, respectively. **D** Phylogenetic tree of the twelve mammalian species and divergence time estimation. MYA, million years ago. A color code was used to highlight species showing partial DNA methylation (brown) as opposed to hypomethylation patterns (cyan)

To investigate conservation of *KBTBD6* promoter methylation in mammals, we compared gametic and/or tissue methylation among 12 mammalian species. Regarding gametic methylation, in humans, non-human primate (rhesus monkey), and mice, the *KBTBD6* promoter CpG island was hypo- or un-methylated in both oocytes and sperm (Fig. 3A). Whereas, in pigs and cows, the CpG island was methylated in oocytes, but not in sperm. Regarding tissue methylation, in humans, non-human primates (crab-eating macaque, chimpanzee, rhesus monkey, and gibbon), and mice, the *KBTBD6* CpG island was hypo- or un-methylated in various fetal and/or adult tissues (Fig. 3B). In contrast, the CpG island was partially methylated in livestock species (horses, pigs, cows, sheep, and goats) and dogs. Lengths of the *KBTBD6* promoter CpG islands tended to be longer in livestock species and dogs, whereas distribution of the length was similar not only across genomes but also in chromosomes containing *KBTBD6* across species, except for mice (mm39 and chromosome 14) that showed an enriched length between 500 and 100 bp (Additional file 4: Supplementary Fig. 8A and B, and Additional file 5: Supplementary Table 4). It appeared that the longer CpG islands in the *KBTBD6* promoter is not a general feature but rather specific to this locus. In addition, through de novo motif discovery followed by motif comparison, we identified unique DNA motifs in the putative *KBTBD6* promoter regions of livestock and dogs and most of them were not matched with databases (Additional file 4: Supplementary Fig. 8C and Additional file 6: Supplementary Table 5). Moreover, multiple alignment of amino acid sequences of KBTBD6 proteins revealed that an ATG8 family-interacting motif (W-V-R-V) in human KBTBD6 is conserved in all analyzed non-human primates, but the R residue (R670) was substituted in all analyzed livestock and dogs (W-V-Q-V) along with other substitutions occurred throughout the residues (Additional file 4: Supplementary Fig. 8D). To determine whether the partial DNA methylation is allelic, we examined reads overlapping the partially methylated domains (PMDs). These PMDs were allelically methylated regions (more than 30% of both hypomethylated and hypermethylated reads) and CpGs in these reads were either fully methylated or unmethylated supporting the presence of allele-specific methylation (Fig. 3C). To examine divergence of the twelve placental mammalian species, we explored the TimeTree and the phylogenetic tree showed divergence *circa* 94 million years ago (MYA) of the following two clades: clade 1 [crab-eating macaque (*Macaca fascicularis*), rhesus monkey (*Macaca mulatta*), chimpanzee (*Pan troglodytes*), human (*Homo sapiens*), gibbon (*Nomascus leucogenys*), and mouse (*Mus musculus*)] and clade 2 [pig (*Sus scrofa*), sheep (*Ovis aries*), goat (*Capra hircus*), cow (*Bos taurus*), horse (*Equus caballus*), and dog (*Canis lupus familiaris*)] (Fig. 3D). In summary, the *KBTBD6* promoter methylation appeared to be monoallelic or maternal-specific in analyzed livestock species and dogs which were diverged from humans, non-human primates, and mice.

### Bi- or mono-allelic expression of the *KBTBD6* gene is grouped in the same manner with the species-specific methylation pattern

**Fig. 4:**
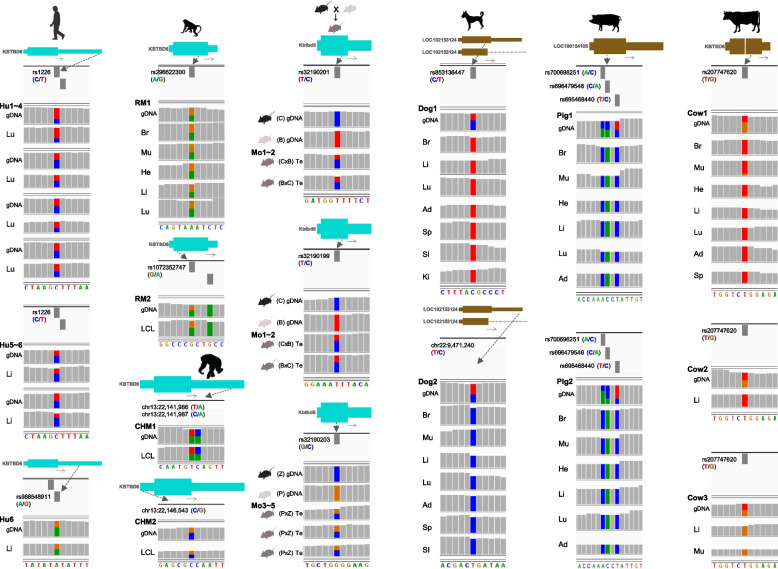
Allelic expression of the *KBTBD6* gene. Heterozygous (i.e., informative) SNPs are shown on genomic DNA (gDNA) except mice which were crossed. SNPs without identifiers (rs IDs) are indicated with genomic coordinates. In the human (Hu), heterozygous SNPs in the normal lung from four individuals [individual IDs from deposited datasets in Additional file 1: Supplementary Table 1 (hereafter, IDs): N3, N16, N26, and N31] and heterozygous SNPs in the normal liver from two individuals (IDs: RK130 and RK141) and one individual (ID: RK141) were analyzed. For rhesus monkeys (RM) two different SNPs in tissues were analyzed (RM1, WGS run ID: SRR1636014 and RNA-seq from the same individual; and RM2, ID: R05040). For chimpanzees (CHM), three different SNPs in tissues were analyzed (IDs: CH114 and CH391). For the mouse (Mo), testes of offspring from reciprocal crosses of CAST/EiJ (C) and C58BL/6 J (B) (RNA-seq run IDs: SRR823506 and SRR823507) and crosses of CZECHII/EiJ (Z) and PWK/PhJ (P) (RNA-seq run IDs: SRR2060844, SRR2060846, and SRR2060939) were analyzed. For dogs, two Belgian Malinois dogs (IDs: Dozer and Crak) were analyzed for two different SNPs. For pigs, three different SNPs from two pigs were analyzed for various tissues (WGS run IDs: SRR7903780 and SRR7903782; and RNA-seq from the same individuals). For cows, the same SNP from three different cows was analyzed (IDs: 6819, 756, and 3847). Lu, lung; Li, liver; Br, brain; Mu, skeletal muscle; He, heart; LCL, lymphoblastoid cell line; Te, testis; Ad, adipose tissue; Sp, spleen; SI, small intestine; Ki, kidney

Bi- or mono-allelic expression of the *KBTBD6* gene was examined using an individual-matched genomic DNA sequence from genome/exome sequencing and mRNA transcripts from RNA-seq. In humans and non-human primates (rhesus monkeys and chimpanzees), heterozygous informative SNPs were found in genomic DNA covering the *KBTBD6* gene and their biallelic expression tendency was repeatedly observed in multiple individuals (Fig. 4 and Additional file 7: Supplementary Table 6). In mice, inbred strains CAST/EiJ (abbreviated as C) and C58BL/6 J (abbreviated as B) that were reciprocally crossed were analyzed. Their offspring expressed both paternal and maternal alleles in testis (Fig. 4 and Additional file 7: Supplementary Table 6, where *Kbtbd6* is predominantly expressed in the analyzed dataset (SRP020526) (Additional file 4: Supplementary Fig. 9) and also in bioGPS based on GSE10246 [78, 79]. This biallelic expression pattern was also observed in crosses of other inbred strains, CZECHII/EiJ (abbreviated as Z) and PWK/PhJ (abbreviated as P) (Fig. 4 and Additional file 7: Supplementary Table 6), as well as in other SNPs in the same offspring (Additional file 4: Supplementary Fig. 10A–C and 11A–C). With the same data, both known maternal and paternal expressions could be detected, depending on the presence of SNPs, in maternally expressed 3 (*Meg3*) (Additional file 4: Supplementary Fig. 10 and 11 left), and paternally expressed 10 (*Peg10*) (Additional file 4: Supplementary Fig. S10 right) or paternally expressed 6 (*Peg6*, aka. *Ndn*) (Additional file 4: Supplementary Fig. 11 right), indicating that both paternal and maternal epigenetic marks were present in the somatic parts of testes and thereby supporting the biallelic expression of *Kbtbd6* without those epigenetic marks. On the other hand, in the same individuals of dogs, pigs, and cows, analyzed heterozygous SNPs were expressed mono-allelically in all analyzed tissues (Fig. 4 and Additional file 7: Supplementary Table 6), suggesting that these monoallelic expressions in livestock species and dogs are attributed to the aforementioned species-specific oocyte methylation and allelic methylation at the *KBTBD6* promoter CpG islands.

### Transcription, histone modifications and DNA methylation at the *KBTBD6* locus occurred in a species-specific manner

**Fig. 5:**
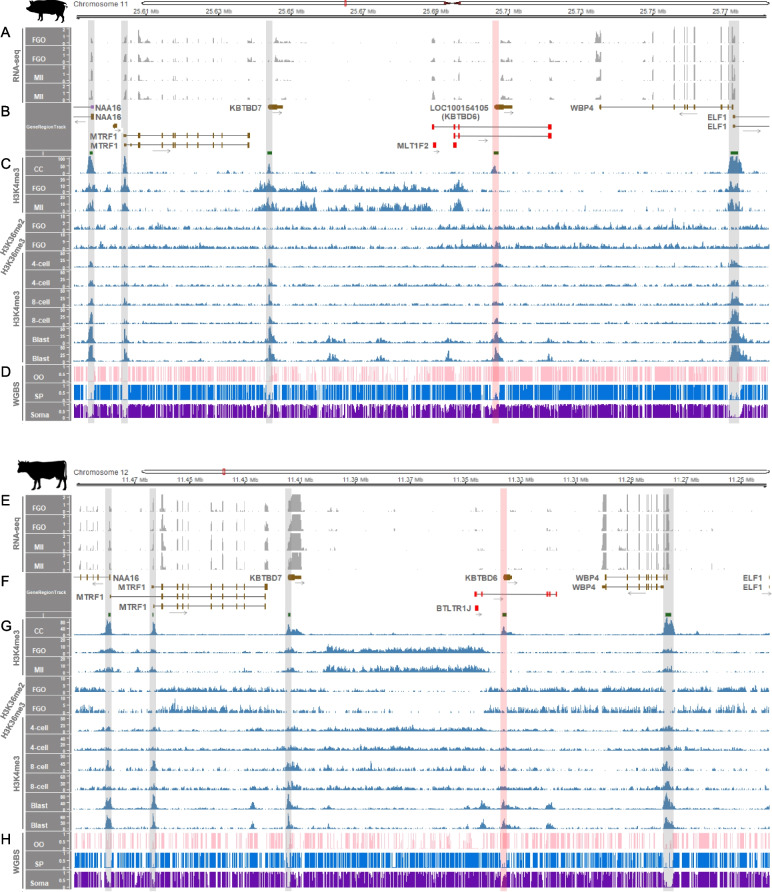
Transcription-dependent imprinting at the CpG island promoter of the *KBTBD6* gene in porcine and bovine oocytes. **A** Expressed transcripts within the locus between the *MTRF1* and *WBP4* genes in porcine oocytes. TPM values of RNA-seq read coverages are depicted. **B** Annotated protein-coding and noncoding transcripts from the susScr11 porcine genome and LTR-initiated transcript in brown, purple, and red colors, respectively, with black arrows for transcriptional directions in GeneRegionTrack based on RefSeq. LTR-RTs [MLT1F2 in + strand (left) and a potential single-copy oocyte-specific promoter region (right)] and CpG islands (I) are color-coded in red and green, respectively. **C** Histone modifications, H3K4me3 for active promoters and H3K36me2/3 for de novo DNA methylation. Data were normalized to 1 × depth (reads per genome coverage, RPGC). **D** Methylation ratios based on WGBS data. **E** Expressed transcripts within the locus between the *MTRF1* and *WBP4* genes in bovine oocytes. RNA-seq read coverages are presented as TPM values. **F** Annotated protein-coding transcripts from the bosTau9 bovine genome and an LTR-initiated transcript in brown and red colors, respectively, with transcriptional directions in black arrows. A bovine-specific LTR-RT [BTLTR1J in – strand; Of note, the bovine chromosome 12 is depicted backwards as indicated in the genomic coordinates (11,470,000 – 11,250,000) due to the reversed orientation of the bovine *KBTBD6* gene.] and CpG islands (I) are color-coded in red and green, respectively. **G** H3K4me3 and H3K36me2/3 modifications normalized to 1 × depth (RPGC). **H** Methylation ratios from WGBS. The promoter regions are highlighted with grey vertical shades. The red vertical shades highlight the CpG island promoters of the *KBTBD6* genes which are methylated in oocytes. FGO, fully grown oocytes; MII, MII stage oocytes; CC, cumulus cells; 4-cell, 4-cell stage embryos; 8-cell, 8-cell stage embryos; Blast, blastocysts; OO, oocytes; SP, sperm; Soma, somatic tissue (skeletal muscle)

Insertion of long terminal repeat retrotransposons (LTR-RTs) in genomes drives a significant amount of transcription, and their presence at orthologous regions is highly variable across species resulting in species-specific transcription in mammalian oocytes and tissues [39]. In addition, H3K36me3 and H3K36me2 deposition at CpG islands embedded within oocyte-specific transcripts precedes DNMT3A/3L-dependent de novo DNA methylation during oogenesis [39]. Comparing pigs, cows, humans, and mice, we aimed to identify species-specific genic and intergenic transcripts within or near the *KBTBD6* locus, initiating in an LTR-RT. Also, to examine whether expressed transcripts at the *KBTBD6* locus in oocytes are related with DNA methylation, omics data (transcriptomes, ChIP-seq data, and methylomes) were analyzed. In fully-grown oocytes (FGOs) and MII stage oocytes from pigs, transcripts might initiate adjacent to a LTR element (MLT1F2 from ERVL-MaLR family) or a potential single-copy oocyte-specific promoter region (as seen in a novel, non-LTR single-copy promoter region in the *Impact* locus active in rat oocytes [80]) in the upstream of *KBTBD6* and cover the promoter CpG island of *KBTBD6* as overlapping transcripts (Fig. 5A and B). These LTR elements might be active in oocytes as being present along with oocyte-specific H3K4me3 enrichment, which was absent in somatic cumulus cells and developing 4-cell, 8-cell, and blastocyst stage embryos (Fig. 5C). Consequently, the active LTR-initiated transcription (LIT) likely resulted in H3K36me2 and H3K36me3 enrichment and de novo DNA methylation overlapping the promoter CpG island of *KBTBD6* in porcine oocytes (Fig. 5C and D). The oocyte-specific co-occurrence of H3K4me3 enrichment, LIT, H3K36me2/3 enrichment, and DNA methylation occurred solely in the upstream of the *KBTBD6* gene and was absent in other promoter regions of the *NAA16*, *MTRF1*, *KBTBD7*, *WBP4* and *ELF1* genes (Fig. 5A–D)*.* Similarly, in bovine FGO and MII stage oocytes, transcription was initiated adjacent to a bovine-specific LTR element (BTLTR1J from ERVK family) in the upstream of *KBTBD6* and spanned the promoter CpG island of *KBTBD6* as an overlapping transcript (Fig. 5E and F). The H3K4me3 enrichment in oocytes suggested activation of the LTR element (Fig. 5G). H3K36me2 and H3K36me3 enrichments and DNA methylation covered the promoter CpG island of *KBTBD6* (Fig. 5G and H). The upstream regions and CpG island promoters of the bovine *NAA16*, *MTRF1*, *KBTBD7*, *WBP4* and *ELF1* genes were not enriched with additional transcripts related to those histone modifications and DNA methylation (Fig. 5E–H).

**Fig. 6:**
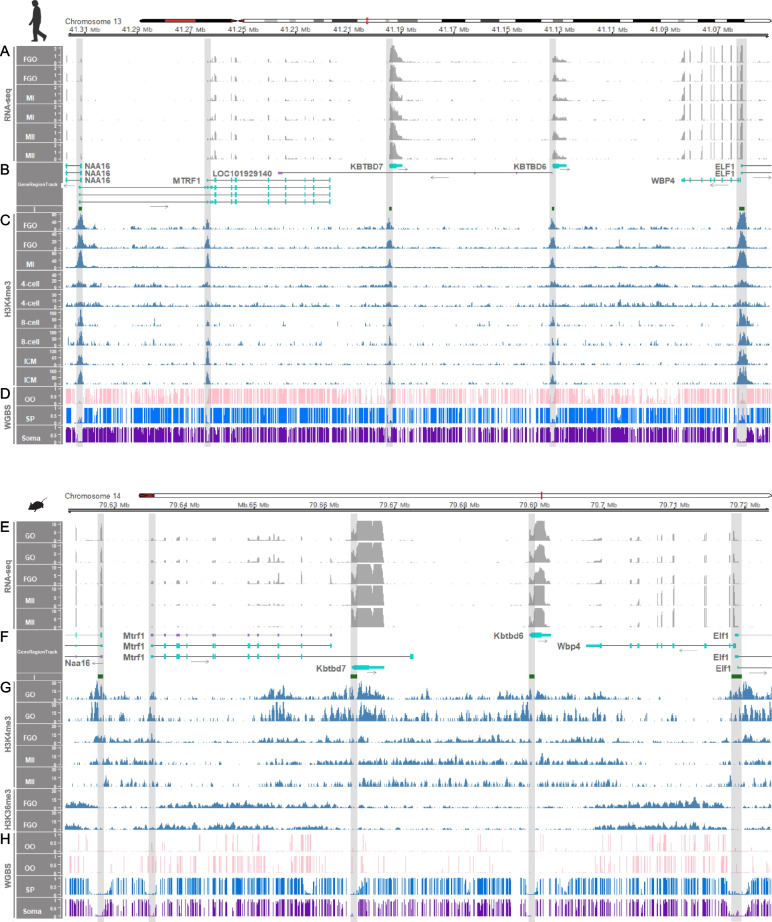
Non-imprinting at the CpG island promoter of the *KBTBD6* gene in human and mouse oocytes. **A** Expressed transcripts from the locus between the *MTRF1* and *WBP4* genes in human oocytes. TPM values of RNA-seq read coverages are presented on the *y*-axis. **B** Annotated protein-coding and noncoding transcripts from the hg38 human genome in cyan and purple colors, respectively. Transcriptional directions are denoted with black arrows. **C** Histone modifications, H3K4me3, for different developmental stages of oocytes and embryos. Data were normalized to 1 × depth (RPGC). **D** Methylation ratios derived from WGBS. **E** Expressed transcripts from the locus between the *Mtrf1* and *Wbp4* genes in C57BL/6N mouse oocytes. TPM values of RNA-seq read coverages are presented. **F** Annotated protein-coding and noncoding transcripts from the mm39 mouse genome in cyan and purple colors, respectively. Transcriptional directions are denoted with black arrows. **G** Histone modifications, H3K4me3 and H3K36me3, normalized to 1 × depth (RPGC). For different developmental stages of oocytes, H3K4me3 modifications from C57BL/6N strain were analyzed. H3K36me3 from C57BL/6 J (top) and CAST/EiJ (bottom) strains were analyzed. **H** Methylation ratios from WGBS of mouse oocytes from C57BL/6 J (top) and CAST/EiJ (bottom) strains. GO, growing oocytes; FGO, fully grown oocytes; MI, MI stage oocytes; MII, MII stage oocytes; 4-cell, 4-cell stage embryos; 8-cell, 8-cell stage embryos; ICM, inner cell mass of the blastocyst; OO, oocytes; SP, sperm; Soma, somatic tissue (liver)

On the other hand, in human oocytes, there was no transcription initiation along with H3K4me3 enrichment in the upstream of *KBTBD6* and transcripts overlapping the promoter CpG island of *KBTBD6* were not found (Fig. 6A–C). H3K4me3 enrichments were concentrated on the promoter regions of the *KBTBD6* gene, as well as all other genes at this locus, i.e., the *NAA16*, *MTRF1*, *KBTBD7*, *WBP4*, and *ELF1* genes (Fig. 6C). Without overlapping transcripts, the promoter CpG islands of the *KBTBD6* gene in oocytes were unmethylated like those of the other genes (Fig. 6D). Also, in mouse oocytes, the promoter of CpG island of *Kbtbd6* was not overlapped by additional transcripts (Fig. 6E and F). This could be due to non-activation of transcription initiation, although the H3K4me3 deposition additionally occurs in intergenic regions to some extent (noncanonical pattern) (Fig. 6G). As previously reported [43], this noncanonical H3K4me3 was observed only in mice, but not in the human in which the H3K4me3 deposition concentrated on the promoter regions (Fig. 6C). Besides the noncanonical pattern, unmethylation at the *KBTBD6* promoter CpG island was conserved in mice (Fig. 6H). The non-deposition of H3K36me3 at the *KBTBD6* promoter CpG island in oocytes supported the unmethylation pattern (Fig. 6G and H). Additionally, in rhesus monkey MII oocytes, the long non-coding RNA (lncRNA) transcript (LOC106994239) was expressed between *KBTBD7* and *KBTBD6* with transcriptional direction toward *KBTBD7*, whereas transcripts overlapping the promoter of *KBTBD6* was apparently absent with unmethylation states (Additional file 4: Supplementary Fig. 12). Furthermore, in the rat oocytes, transcriptional initiation at the promoter of *Kbtbd6* lacked as being deficient in another rodent species, mice (Additional file 4: Supplementary Fig. 13).

Taken together, the initiation patterns of porcine and bovine transcripts in oocytes along with hypermethylation and H3K36me3 and H3K36me2 enrichments were similar, suggesting evolutionary conservation of transcription-dependent de novo DNA methylation at the *KBTBD6* locus. This conservation of imprinting in porcine and bovine species might be diverged from non-imprinting of *KBTBD6* in primates and mice.

### Phylogenetic representation indicates a recent acquisition of *KBTBD6* imprinting

**Fig. 7:**
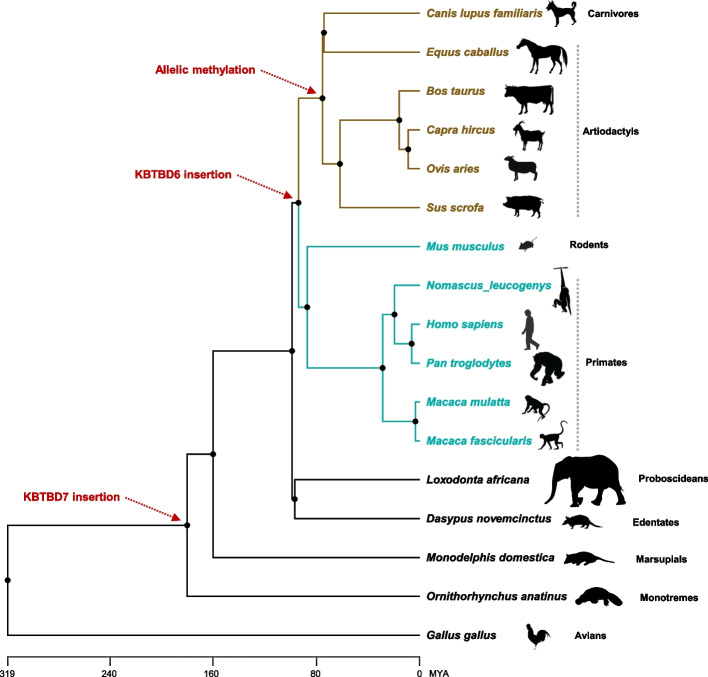
Phylogenetic relationships of amniotes. The *KBTBD7* and *KBTBD6* gene insertions are indicated with red dotted arrows. Allelic methylation of the *KBTBD6* promoter is denoted with a red dotted arrow for species of artiodactyls and carnivores in brown color. Unmethylation of the *KBTBD6* promoter was shown for species of primates and rodents in cyan colors. MYA, million years ago. Silhouette images of species are from phylopic.org

Schematic representation exhibited that *KBTBD7* and *KBTBD6* gene insertions occurred approximately 180 and 94 MYA, respectively (Fig. 7). In chickens (*Gallus gallus*), the whole locus between *KBTBD7* and *KBTBD6* is deficient while the surrounding *MTRF1* and *WBP4* genes exist based on NCBI RefSeq annotation. In platypus (*Ornithorhynchus anatinus*), opossum (*Monodelphis domestica*), armadillo (*Dasypus novemcinctus*), and elephant (*Loxodonta africana*), genes are in the order of *MTRF1*, *KBTBD7*, and *WBP4* with undefined sequences between *KBTBD7* and *WBP4* in cases of armadillo and elephant. The *KBTBD6* insertion resulted in two types of promoter methylation: unmethylation in humans, non-human primates, and mice, and allelic methylation in analyzed domesticated mammals as shown in this study (Fig. 3). The allelic methylation might be introduced in approx. 75 MYA in artiodactyls and carnivores (Fig. 7). The expression of non-imprinted *KBTBD6* genes in humans, non-human primates, and mice was biallelic. In analyzed domesticated mammals, imprinted monoallelic expression appeared to be selectively evolved. In this sense, the notion that the epigenetic fate of genes can be dependent on selective forces at the sequence integration site could be supported by our findings on porcine and bovine-specific transcription-dependent imprinting of the *KBTBD6* gene (Fig. 5).

## Discussion

In this study, we report that the imprinted *KBTBD6* gene in pigs is subjected to a direct silencing of the maternal allele of its promoter region through maternal DNA methylation. This silencing could lead to lack of expression of the porcine *KBTBD6* gene in the bi-maternal PA embryos. Because the paternal allele is present only in CN embryos, but not in PA embryos, the exclusive *KBTBD6* expression in the CN embryos was likely to be paternal allele-specific. These findings were effectively derived from generation of replicated PA and CN embryos followed by a combined analysis of the single base-resolution methylome and transcriptome which enabled detailed imprinting studies both globally at a genomic level and within targeted loci.

As mentioned, the regulation of *RB1* expression in humans through intronic maternal methylation [73, 74] was not conserved in pigs. Also, our comparative analyses revealed that the putative promoter region of the *KBTBD6* gene is methylated in a species-specific manner: allelic methylation in analyzed domesticated mammals [livestock species (horses, pigs, cows, sheep, and goats) and dogs]; whereas unmethylated in humans, non-human primates, and mice. In accordance with the methylation pattern, monoallelic expression of the *KBTBD6* gene was observed in pigs, cows, and dogs, while the *KBTBD6* gene was biallelically expressed in humans, rhesus monkeys, chimpanzees, and mice. Therefore, the bi- or mono-allelic expression pattern of the *KBTBD6* gene exhibited the same species-specificity as allelic methylation in analyzed species, although limitation was whether the monoallelic expression was paternal or maternal origin could not be identified with the analyzed datasets of WGS/exome-seq and RNA-seq from the same offspring without parental information. However, in our parthenogenesis approach, CN embryos have both maternal and paternal alleles and PA embryos have two maternal alleles, and therefore, exclusive expression of *KBTBD6* in CN embryos could be interpreted as paternal monoallelic expression. This expression in CN embryos occurred with unmethylation of the promoter in the paternal allele of CN embryos, as evidenced by the DMR that was hypermethylated in PA embryos (i.e., bi-maternal methylation) and hemi-methylated in CN embryos (i.e., uni-maternal methylation).

The establishment of maternal methylation imprints in the oocytes occurs within transcribed regions enriched with H3K36me3 and H3K36me2 histone modifications via recruitment of DNMT3A and 3L [81–83]; whereas, paternal imprints marked with H3K36me2 locate in non-transcribed intergenic regions [43, 81, 84, 85]. DNA methylation imprints in the promoter region of the *KBTBD6* gene in pig and cow oocytes also occurred with additional transcription and H3K36me2/3 marks, suggesting these are maternal imprints, which were not found in humans, non-human primates, and mice. The phylogenetic analysis showed that the separation between one clade of humans, non-human primates, and mice and another clade of livestock species and dogs coincidently matches with allelic DNA methylation patterns in the *KBTBD6* promoter CpG islands. We postulate that the *KBTBD6* promoter CpG islands are likely to be one of the loci in which evolutionary pressure operated selectively between the two clades.

Genomic insertions of transposable elements (Tes), such as LTR retrotransposons, give rise to initiation of gene transcription which are active in germ cells [39]. Eukaryotic Tes can insert anywhere in the host genome while contributing to the evolution of transcriptional regulation and gene expression, DNA methylation, and genomic instability [86]. Among the four classes of Tes which include three types of retrotransposons, genomic insertion of LTR retrotransposons comprises about 9% of mammalian genomes [87–89] and their influences on transcription initiations have been reported [39, 90]. In this regard, species-specific LTR-initiated transcription could shape the oocyte methylome, as CpG islands in promoters are embedded within transcripts and methylated as in the cases including *Impact* and *Slc38a4* in mice [39, 80]. In pigs and cows, potential transcripts were initiated within or adjacent to LTR-RTs, upstream of the *KBTBD6* gene in oocytes. In contrast, in mice and humans, no additional transcription and corresponding Tes were observed other than the relatively prominent *KBTBD6* expression. Together with the distinctive transcription in pigs and cows, the chromatin status under H3K36me3 and H3K36me2 histone modifications might help direct de novo DNA methylation at the transcribed loci. As a consequence, maternal imprinted germline DMRs (igDMRs) in oocytes were heavily methylated under this methylation-permissive state, which was not the case of mice and primates.

The paternal expression of *KBTBD6* might be related to growth traits following the parental conflict theory that states paternally expressed genes promote growth; whereas, maternally expressed genes are related to reduced growth [91, 92]. Based on the pig quantitative trait loci (QTL) database (PigQTLdb) [93] and a related report [94], there is information regarding QTLs mapping with the porcine *KBTBD6* locus in association with backfat weight (QTL #422), percentage of backfat and leaf fat in the carcass (QTL #423), and backfat thickness between the 3^rd^ and 4^th^ rib (QTL #425). Also, based on the cattle QTL database (CattleQTLdb) [93] and a related report [95], a QTL associated with body weight (weaning) (QTL #4481) was aligned with the bovine *KBTBD6* locus, and further studies are needed to fully assess the role of *KBTBD6* in development and growth. It has been reported that the KBTBD6 protein is involved in the proteasome-mediated ubiquitin-dependent protein catabolic process. In particular, KBTBD6 serves as a substrate adaptor for the aforementioned ubiquitin ligase complex consisting of a dimer of KBTBD6 and KBTBD7 and CUL3 in human cell lines [13, 14]. KBTBD6 contains an ATG8 family-interacting motif (AIM), W-V-R-V, and binds to GABARAP proteins (ATG8 family proteins). For this interaction, the R residue (R670) in the AIM of KBTBD6 is critical as it forms a hydrogen bond with Y25 of GABARAP, and this interaction subsequently leads to proteasomal degradation of TIAM1, a RAC1 activator, and spatially regulated RAC1 signaling [14]. The substitution of R670 of AIM in all analyzed livestock and dogs (W-V-Q-V) suggests destabilization of a protein–protein interaction or conformational changes between the CUL3-KBTBD6/ KBTBD7 ubiquitination complex and GABARAP. Whether this non-conservation of R residue is related to epigenetic reduction of *KBTBD6* gene dosage by half through genomic imprinting is elusive, but future studies on the ubiquitination complex and RAC1 signaling together with genome editing on the imprinting control region will provide more insight into the livestock-specific *KBTBD6* imprinting. Also, a deficiency in *KBTBD6* expression in parthenotes might lead to abnormalities in the generation of the complex and the subsequent RAC1 signaling which affects actin rearrangements. Taken together, our comparative studies revealed that paternal expression of the *KBTBD6* gene in pigs and its monoallelic expression in analyzed livestock and dogs could be related to maternal methylation along with additional gene transcription, indicating locus-specific and non-clustered genomic imprinting at the *KBTBD6* locus. The paternal expression of *KBTBD6* might be related to animal growth, while its biallelic expression in humans, non-human primates, and mice might be diverged during the course of evolution.

## Conclusions

Genomic imprinting is an epigenetic process that causes parent-of-origin-specific monoallelic expression of a subset of genes and is essential for mammalian growth and development. Imprinting research on domesticated animals has focused on imprinted genes previously identified in mice and humans, but, unlike in mice and humans, the porcine *KBTBD6* gene was expressed only from the paternal allele and the *KBTBD6* promoter was encompassed by a DMR with maternal methylation, indicating imprinting of *KBTBD6*. This imprinting is apparently conserved in analyzed domesticated mammals, but not in humans, non-human primates, and mice. We also provide potential mechanistic links between transcription and maternal methylation in porcine and bovine oocytes. Our findings indicate that genomic imprinting at the *KBTBD6* locus is selectively evolved in domesticated mammals.

### Supplementary Information

**Additional file 1: Supplementary Table 1.** Deposited data.

**Additional file 2: Supplementary Table 2.** Analyses of partially methylated domains (PMDs).

**Additional file 3: Supplementary Table 3.** Summary of WGBS and RNA-seq data from PA and CN embryos.

**Additional file 4: Supplementary Fig. 1.** Distribution of DNA methylome produced by WGBS. **Supplementary** **Fig. 2.** The *RB1* locus in the human. **Supplementary** **Fig. 3.** The *PPP1R26* locus in the pig. **Supplementary** **Fig. 4.** The *RB1* locus in the pig. **Supplementary** **Fig. 5.** DNA methylation profile within the human locus between *MTRF1* and *WBP4*. **Supplementary** **Fig. 6.** mRNA expression levels within the *MTRF1*-*WBP4* interval in pig embryos. **Supplementary** **Fig. 7.** Positive and negative controls of maternal imprinting. **Supplementary** **Fig. 8.** Comparison of CpG islands in the promoter region of *KBTBD6* gene and motif analyses. **Supplementary** **Fig. 9.** Mouse *Kbtbd6* mRNA expression. **Supplementary** **Fig. 10.** Biallelic expression of mouse *Kbtbd6* in F1i and F1r. **Supplementary** **Fig. 11.** Biallelic expression of mouse *Kbtbd6* in F1. **Supplementary** **Fig. 12.** Non-transcriptional initiation and unmethylation at the CpG promoter of *KBTBD6* in the rhesus monkey. **Supplementary** **Fig. 13.** Absence of transcriptional initiation at the CpG promoter of the *Kbtbd6* gene in the rat.

**Additional file 5: Supplementary Table 4.** Comparison of CpG island lengths across species.

**Additional file 6: Supplementary Table 5.** Analyses of unique (non-matched) motifs within the putative *KBTBD6* promoters in livestock and dogs.

**Additional file 7: Supplementary Table 6.** Read counts of informative SNPs within the *KBTBD6* transcripts.

## Data Availability

Sequencing data generated in this study have been submitted to the NCBI Gene Expression Omnibus (GEO; https://www.ncbi.nlm.nih.gov/geo/) under accession number GSE218487.

## Abbreviations

CN: Control embryo
DEG: Differentially expression gene
DMR: Differentially methylated region
FDR: False discovery rate
ICR: Imprinting control region
KBTBD6: Kelch repeat and BTB domain-containing protein 6
LIT: LTR-initiated transcription
LTR-RT: Long terminal repeat retrotransposon
MR: Methylated region
MYA: Million years ago
PA: Parthenogenetically activated embryo
QTL: Quantitative trait loci
RNA-seq: RNA sequencing
TSS: Transcription start site
WGBS: Whole-genome bisulfite sequencing
