# Supplementary material for: Imprinting at the KBTBD6 locus involves species-specific maternal methylation and monoallelic expression in livestock animals
Source: J Anim Sci Biotechnol. 2023 Oct 11;14:131. doi: 10.1186/s40104-023-00931-3 (PMC10565993; doi:10.1186/s40104-023-00931-3)
Supplement: Supplementary file 4 — Additional file 4: Supplementary Fig. 1. Distribution of DNA methylome produced by WGBS. Supplementary Fig. 2. The RB1 locus in the human. Supplementary Fig. 3. The PPP1R26 locus in the pig. Supplementary Fig. 4. The RB1 locus in the pig. Supplementary Fig. 5. DNA methylation profile within the human locus between MTRF1 and WBP4. Supplementary Fig. 6. mRNA expression levels within the MTRF1-WBP4 interval in pig embryos. Supplementary Fig. 7. Positive and negative controls of maternal imprinting. Supplementary Fig. 8. Comparison of CpG islands in the promoter region of KBTBD6 gene and motif analyses. Supplementary Fig. 9. Mouse Kbtbd6 mRNA expression. Supplementary Fig. 10. Biallelic expression of mouse Kbtbd6 in F1i and F1r. Supplementary Fig. 11. Biallelic expression of mouse Kbtbd6 in F1. Supplementary Fig. 12. Non-transcriptional initiation and unmethylation at the CpG promoter of KBTBD6 in the rhesus monkey. Supplementary Fig. 13. Absence of transcriptional initiation at the CpG promoter of the Kbtbd6 gene in the rat. [file 40104_2023_931_MOESM4_ESM.pdf]

Imprinting at the *KBTBD6* locus involves species-specific maternal methylation and monoallelic expression

Jinsoo Ahn, In-Sul Hwang, Mi-Ryung Park, Seongsoo Hwang & Kichoon Lee

Supplementary Figure 1. Distribution of DNA methylome produced by WGBS.
Supplementary Figure 2. The *RB1* locus in the human.
Supplementary Figure 3. The *PPP1R26* locus in the pig.
Supplementary Figure 4. The *RB1* locus in the pig.
Supplementary Figure 5. DNA methylation profile within the human locus between *MTRF1* and *WBP4*.
Supplementary Figure 6. mRNA expression levels within the *MTRF1-WBP4* interval in pig embryos.
Supplementary Figure 7. Positive and negative controls of maternal imprinting.
Supplementary Figure 8. Comparison of CpG islands in the promoter region of *KBTBD6* gene and motif analyses.
Supplementary Figure 9. Mouse *Kbtbd6* mRNA expression.
Supplementary Figure 10. Biallelic expression of mouse *Kbtbd6* in F1i and F1r.
Supplementary Figure 11. Biallelic expression of mouse *Kbtbd6* in F1.
Supplementary Figure 12. Non-transcriptional initiation and unmethylation at the CpG promoter of *KBTBD6* in the rhesus monkey.
Supplementary Figure 13. Absence of transcriptional initiation at the CpG promoter of the *Kbtbd6* gene in the rat.

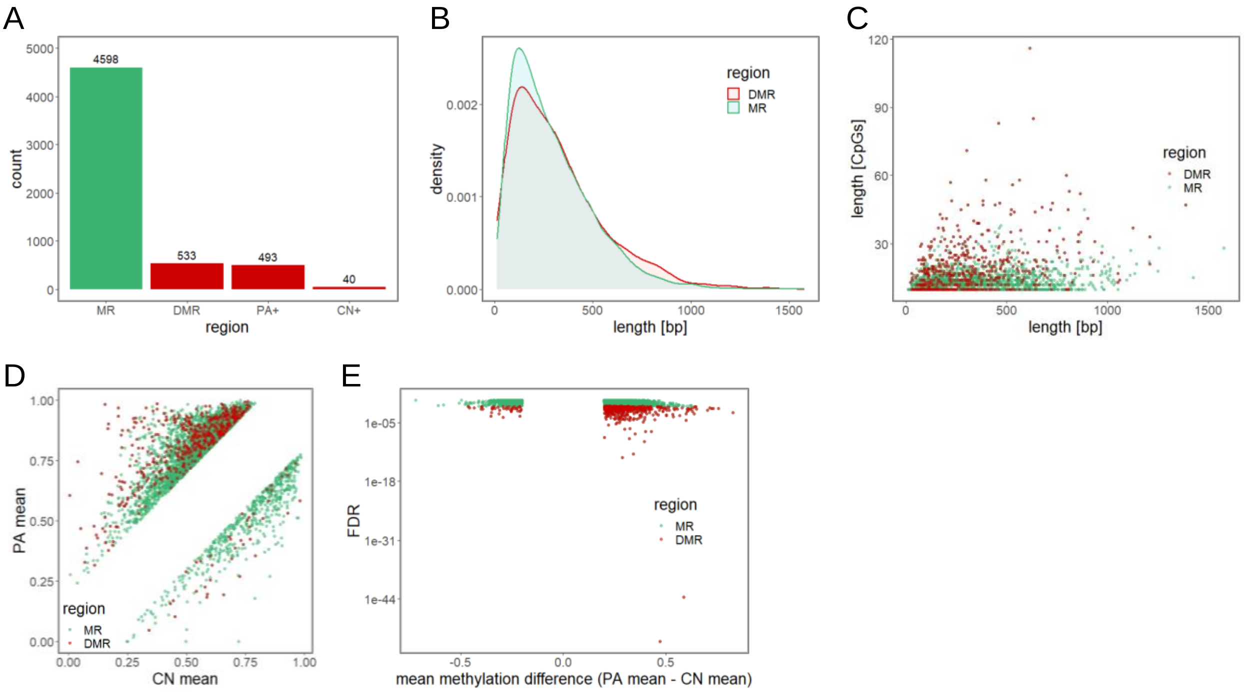

Supplementary Figure 1. Distribution of DNA methylome produced by WGBS. (**A**) A bar plot of regions showed a difference between PA and CN embryos in pig chromosome 11. Methylated regions (MRs) passed criteria of maximum CpG distance of 300 bp, minimum CpG number of 10, and minimum mean methylation difference (PA mean – CN mean) of 0.2. Among MRs, differentially methylated regions (DMRs) satisfied FDR < 0.05. PA+, DMRs hypermethylated in PA embryos; CN+, DMRs hypermethylated in CN embryos. (**B**) A density plot (area under curve = 1) of MR and DMR lengths in base pairs (bps). (**C**) A scatter plot of MR and DMR lengths in bps with CpG dinucleotide lengths. (**D**) A scatter plot of mean methylation levels (with minimum mean methylation difference of 0.2 in the above criteria). (**E**) A dot plot of mean methylation difference between PA and CN embryos against FDR. Below the threshold of 0.05 (5e-02), DMRs are shown with a higher significance level towards the bottom.

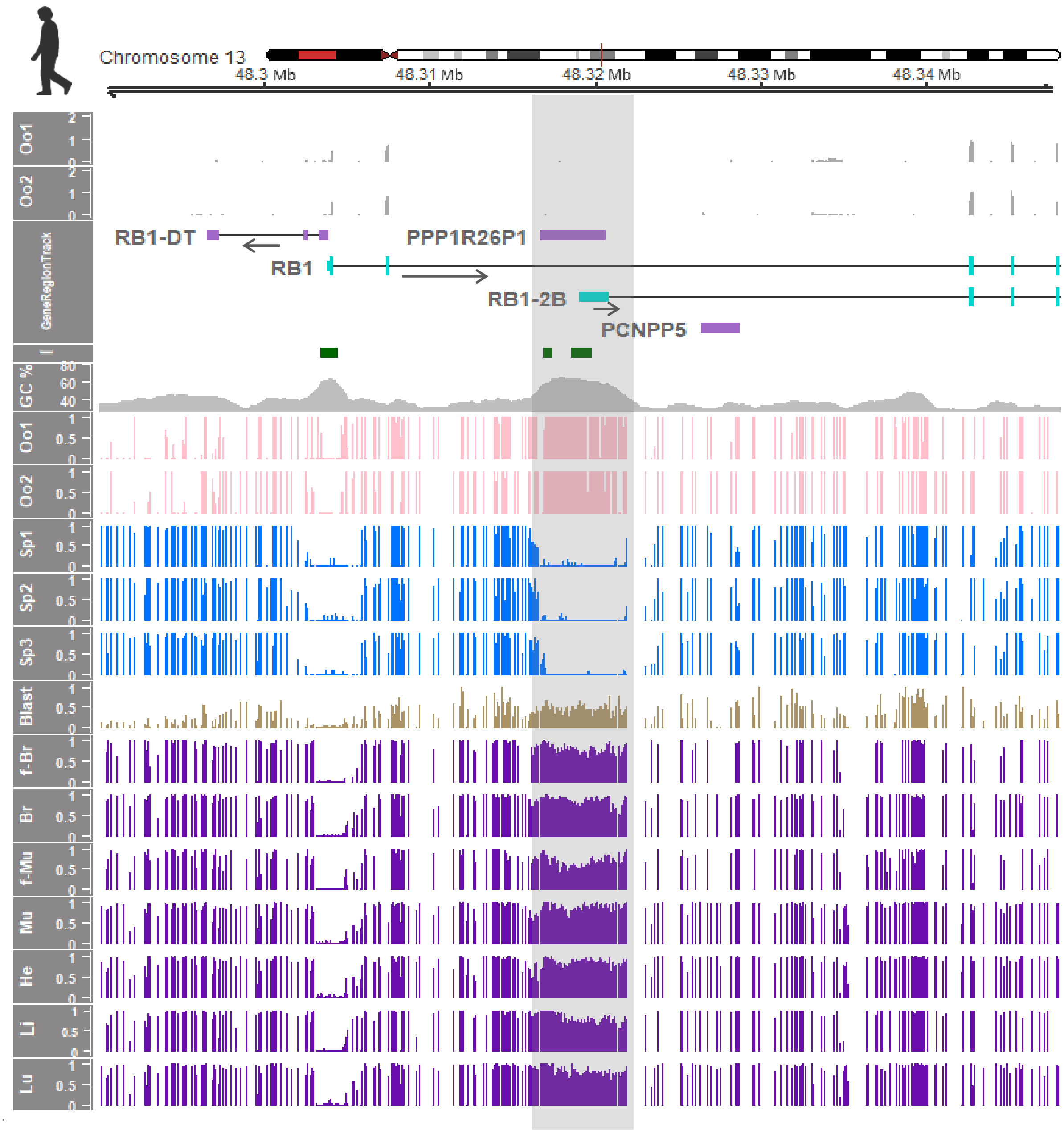

Supplementary Figure 2. The *RB1* locus in the human. DNA methylation in human oocytes (Oo1 ~ 2), sperm (Sp1 ~ 3), blastocyst (Blast) and tissues (f-Br, fetal brain; Br, brain; f-Mu, fetal muscle; Mu, muscle; He, heart; Li, liver; Lu, lung).

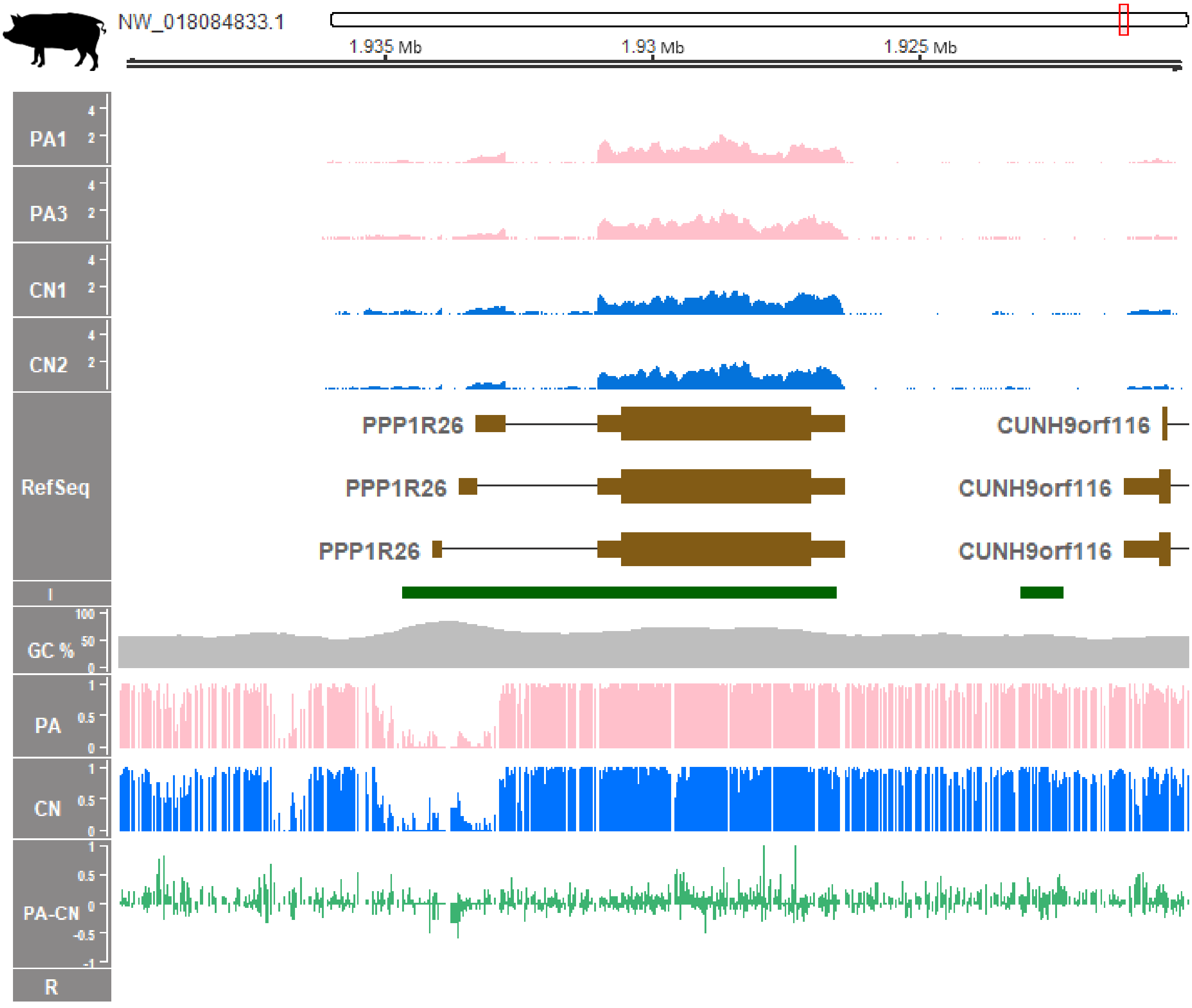

Supplementary Figure 3. The *PPP1R26* locus in the pig. The porcine *PPP1R26* gene is located in the unplaced scaffold (NW_018084833.1) of the current susScr11 genome assembly of the pig.

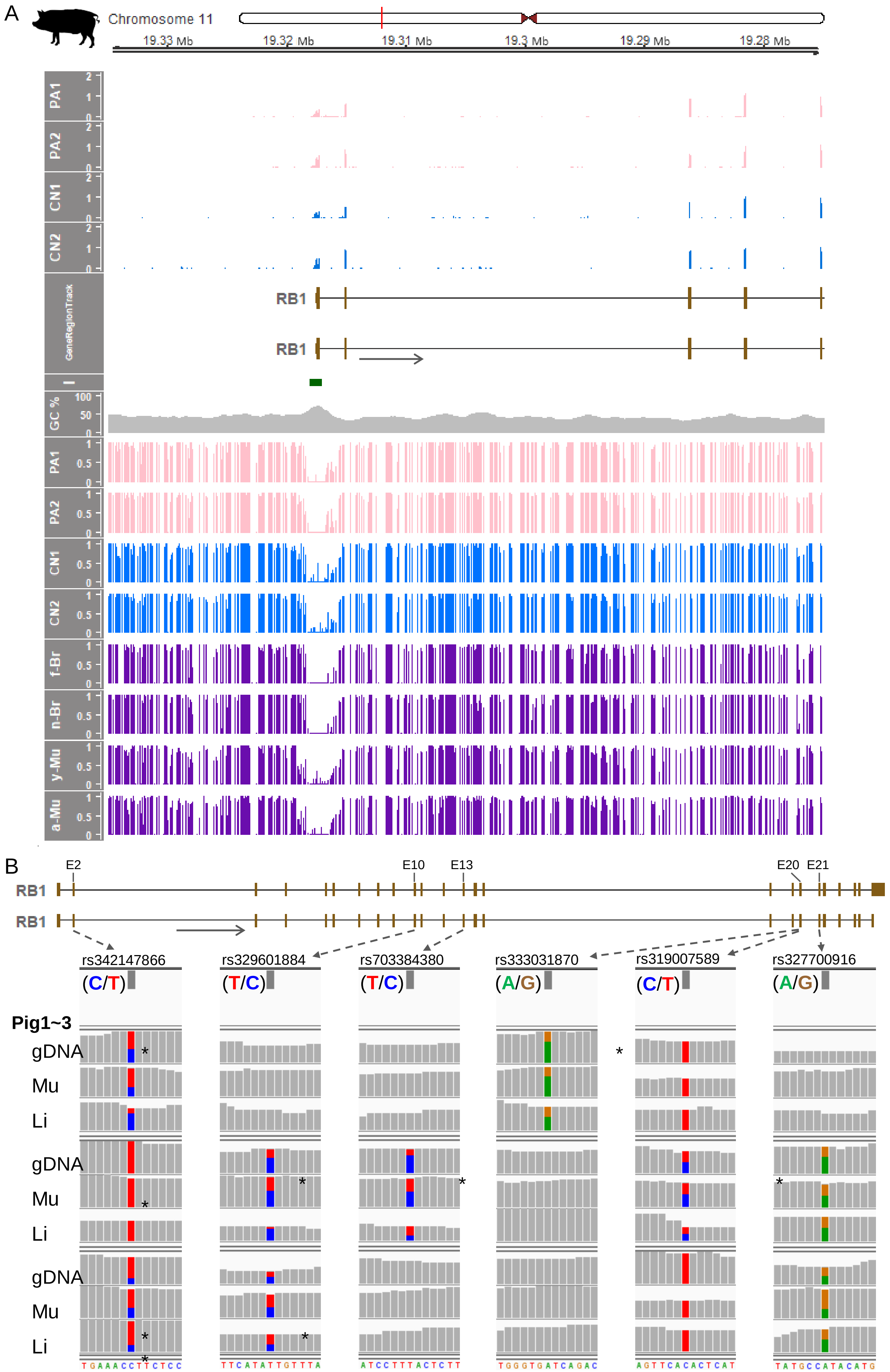

A
Chromosome 11
19.33 Mb
19.32 Mb
19.31 Mb
19.3 Mb
19.29 Mb
19.28 Mb
PA1
PA2
CN1
CN2
GeneRegionTrack
RB1
RB1
GC %
f-Br
n-Br
y-Mu
a-Mu
B
E2
E10
E13
E20
E21
RB1
RB1
rs342147866
(C/T)
rs329601884
(T/C)
rs703384380
(T/C)
rs333031870
(A/G)
rs319007589
(C/T)
rs327700916
(A/G)
Pig1~3
gDNA
Mu
Li
gDNA
Mu
Li
gDNA
Mu
Li
TGAAACCTTCTCC
TTCATATTGTTTA
ATCCTTTACTCTT
TGGGTGATCAGAC
AGTTCACACTCAT
TATGCCATACATG

Supplementary Figure 4. The *RB1* locus in the pig. (**A**) DNA methylation in pig parthenogenetic and normal embryos and pig tissues [f-Br, fetal brain; n-Br, neonatal brain; y-Mu, young muscle (d40); a-Mu, adult muscle (d180)]. (**B**) Allelic expression of the *RB1* transcripts in pig 1 ~ 3 muscle (Mu) and liver (Li).

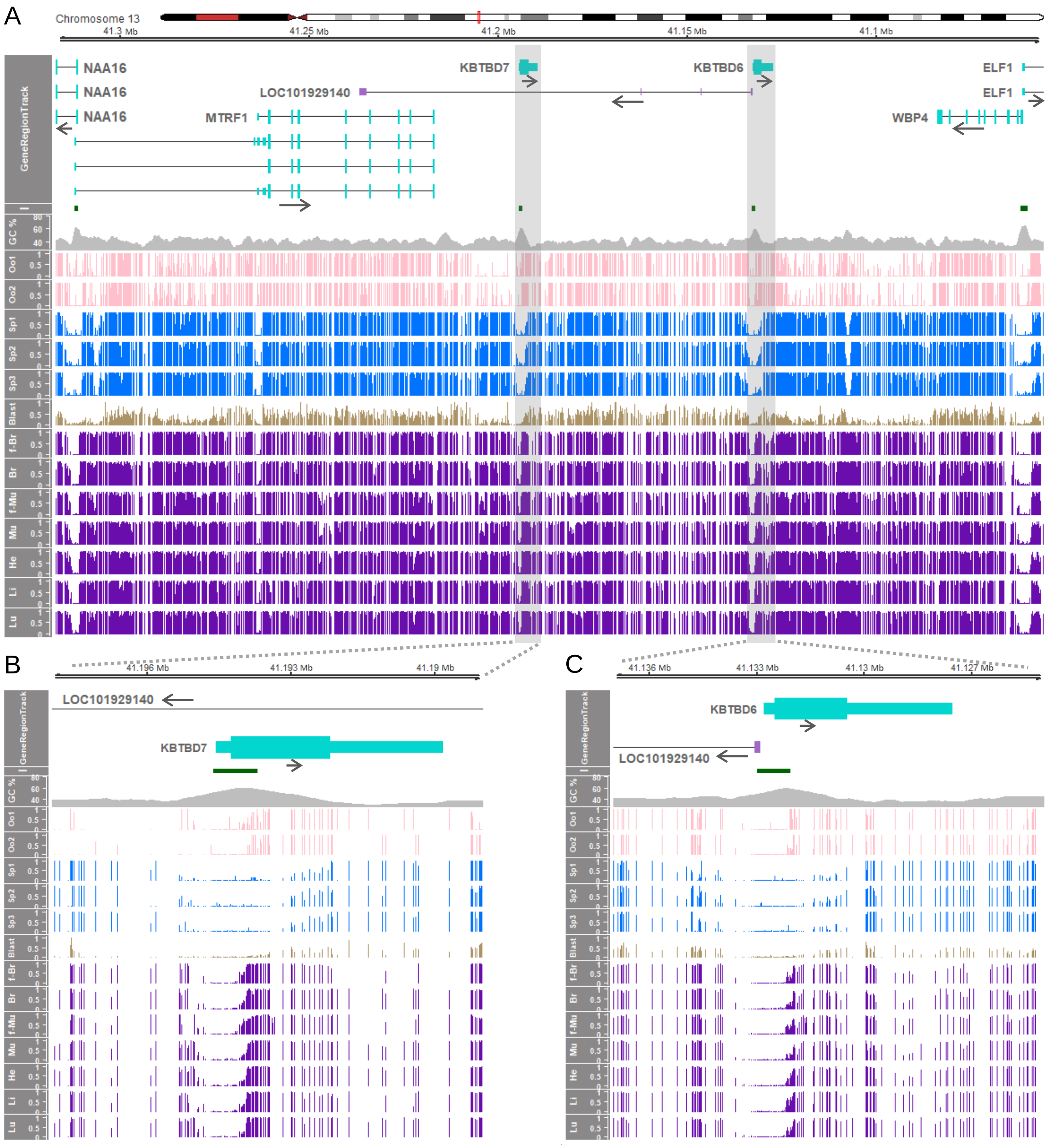

Supplementary Figure 5. DNA methylation profile within the human locus between *MTRF1* and *WBP4*. (**A**) In GeneRegionTrack, transcripts of four protein coding genes (*MTRF1*, *KBTBD7*, *KBTBD6*, and *WBP4*) are displayed with light blue boxes (tall box, translated region; short box, untranslated region). A non-coding transcript (*LOC101929140*) is denoted with purple boxes. The direction of transcription is marked with black arrows. Below GeneRegionTrack, titles on the left panel represent I (CpG island), GC% (GC content), and methylation ratios in Oo (oocyte), Sp (sperm), Blast (blastocyst), f-Br (fetal brain), Br (brain), f-Mu (fetal muscle), Mu (muscle), He (heart), Li (liver), and Lu (lung). The *KBTBD7* and *KBTBD6* genes are zoomed as shown in the two bottom plots (**B,C**). Data of oocytes, sperm, and blastocysts were derived from JGAS00000000006 (hum0009v1.CpG.v1) and data of tissues were from the Human Reference Epigenome Mapping Project datasets (GSE17312).

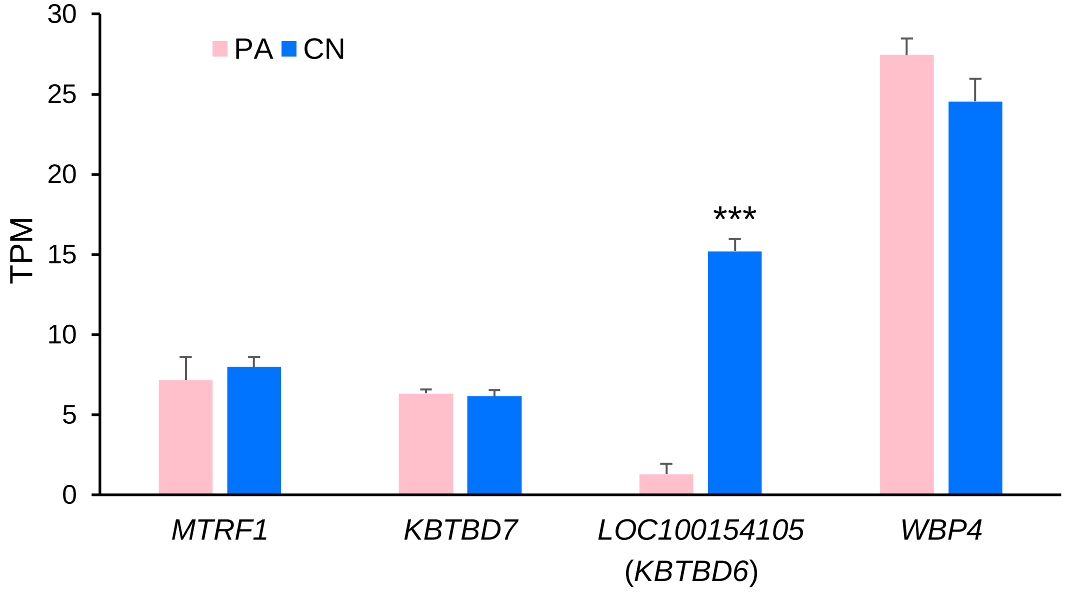

Supplementary Figure 6. mRNA expression levels within the *MTRF1-WBP4* interval in pig embryos. RNA-seq read coverages obtained from whole parthenogenetic (PA) and normal control (CN) embryos were normalized to TPM. Values are presented as mean ± SEM for biological replicates. ***, FDR < 0.001.

A

B

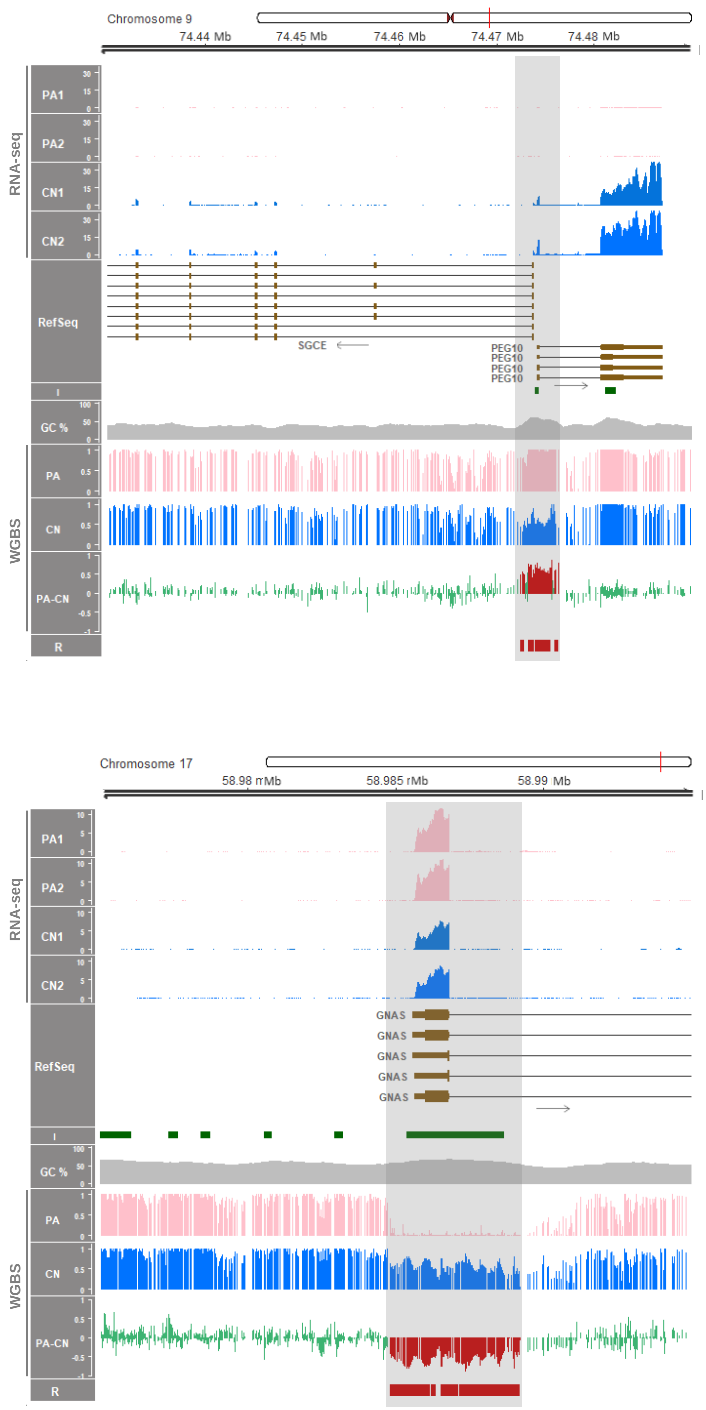

Supplementary Figure 7. Positive and negative controls of maternal imprinting in pig embryos. (A) As a positive control, the known maternally imprinted locus, *SGCE*/*PEG10*, is shown. Their expression was exclusive in CN embryos. A DMR with maternal DNA methylation spans the promoter regions of both *SGCE* and *PEG10*. **(B)** A negative control is exemplified as paternal DNA methylation (paternal imprinting). At the porcine *GNAS* locus, the expression of isoforms of *GNAS* transcripts (also known as *NESP*) was approximately 1.5-fold greater on average in PA embryos. A large DNA methylation canyon and a partial DNA methylation pattern are shown in PA and CN embryos, respectively.

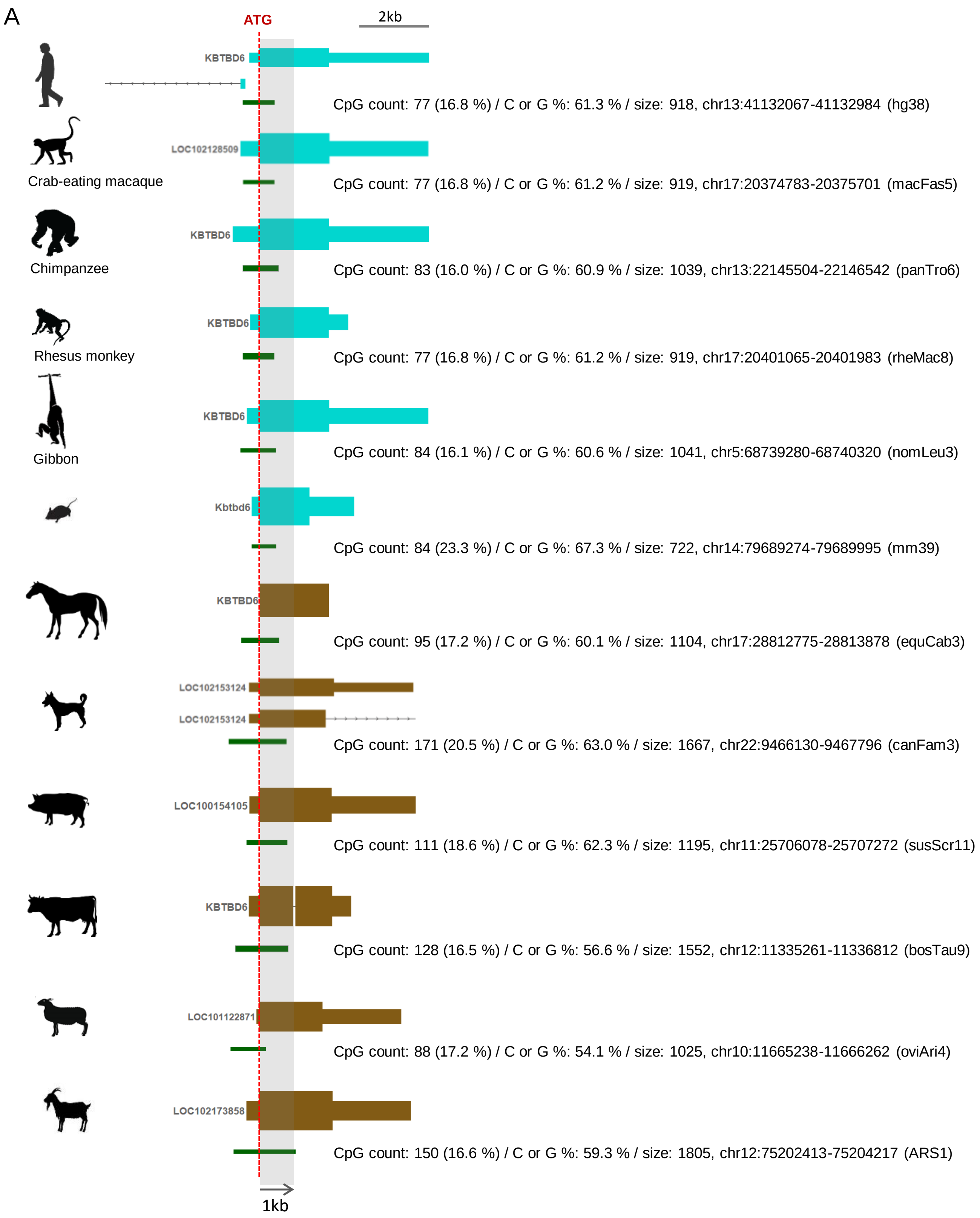

Supplementary Figure 8. Comparison of CpG islands in the promoter region of *KBTBD6* gene and motif analyses. (A) Information of CpG islands was derived from the UCSC genome browser database. Reference genomes for each species are parenthesized at the end. A color code was used to distinguish species displaying DNA hypomethylation (cyan) and partial DNA methylation (brown) in the promoter region of *KBTBD6* as presented in Figure 3.

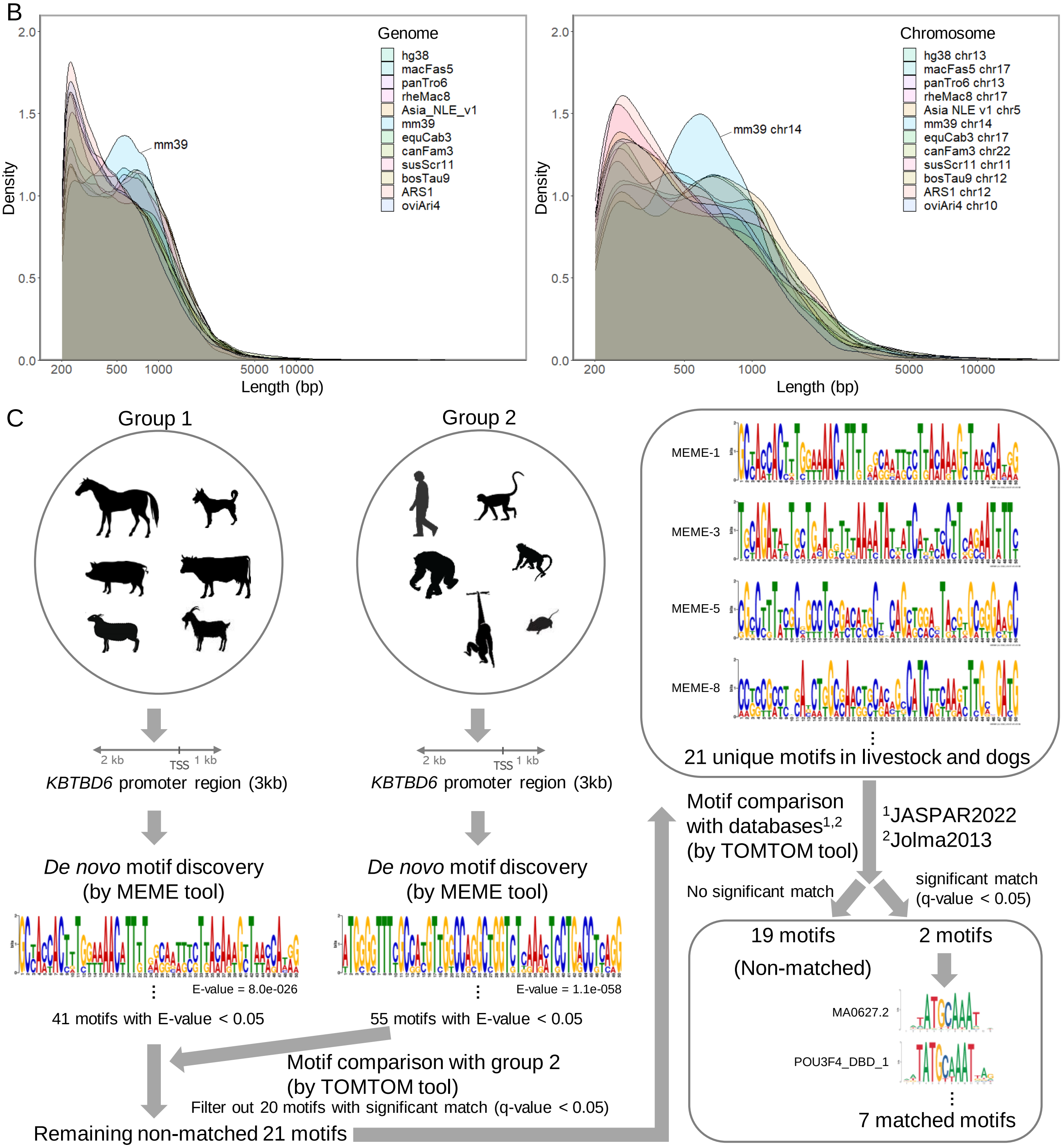

Supplementary Figure 8 (Con'd). (B) Density plots of distribution of the lengths of CpG islands. To draw plots for each genome (left; min, 201 bp; max, 322257 bp) and every chromosome containing the *KBTBD6* gene (right; min, 201 bp; max, 18474 bp), BED format files of CpG islands were downloaded from the UCSC Table Browser (http://genome.ucsc.edu/cgi-bin/hgTables). For gibbon and goat for which BED files are not available, BigBed files were downloaded from the following webpages[3,4] and converted to BED files. Unplaced scaffolds were not included in the plots. **(C)** Motif discovery using the MEME tool (with parameters -mod zoops -maxw 50) from a standalone version of the MEME Suite (v.5.5.3), followed by motif comparison using the TOMTOM tool (with a parameter -dist pearson). A process of identifying 21 non-matched motifs that are unique to livestock and dogs and, among them, 2 matched or 19 non-matched motifs with databases are illustrated, and these motifs are listed in Supplementary Table 5.

[1]JASPAR2022_CORE_vertebrates_non-redundant_v2.meme and [2]EUKARYOTE/jolma2013.meme were downloaded via https://meme-suite.org /meme/meme-software/Databases/motifs/motif_databases.12.23.tgz
[3]https://hgdownload.soe.ucsc.edu/hubs/GCF/006/542/625/GCF_006542625.1/bbi/GCF_006542625.1_Asia_NLE_v1.cpgIslandExt.bb (gibbon)
[4]https://hgdownload.soe.ucsc.edu/hubs/GCF/001/704/415/GCF_001704415.2/bbi/GCF_001704415.2_ARS1.2.cpgIslandExt.bb (goat)

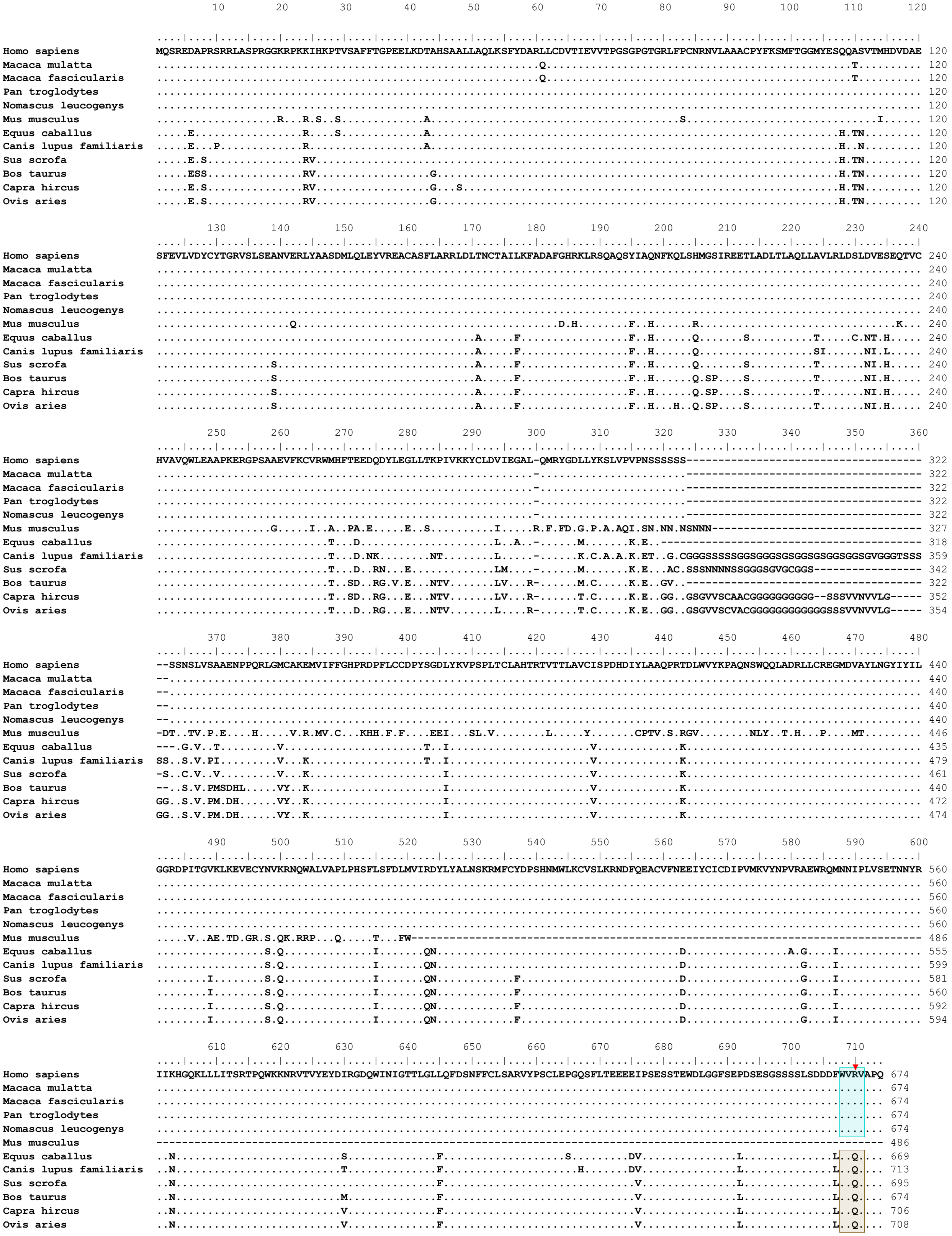

Supplementary Figure 8 (Con'd). (D) Multiple alignment of amino acid sequences of KBTBD6 proteins. The ATG8 family-interacting motifs (W-V-R-V) in humans and non-human primates are enclosed in boxes highlighted with cyan. A corresponding region (W-V-Q-V) in livestock and dogs is highlighted with brown. A red arrowhead indicates the R residue (R670). Dots and hyphens represent identical amino acids and deletions, respectively.

A

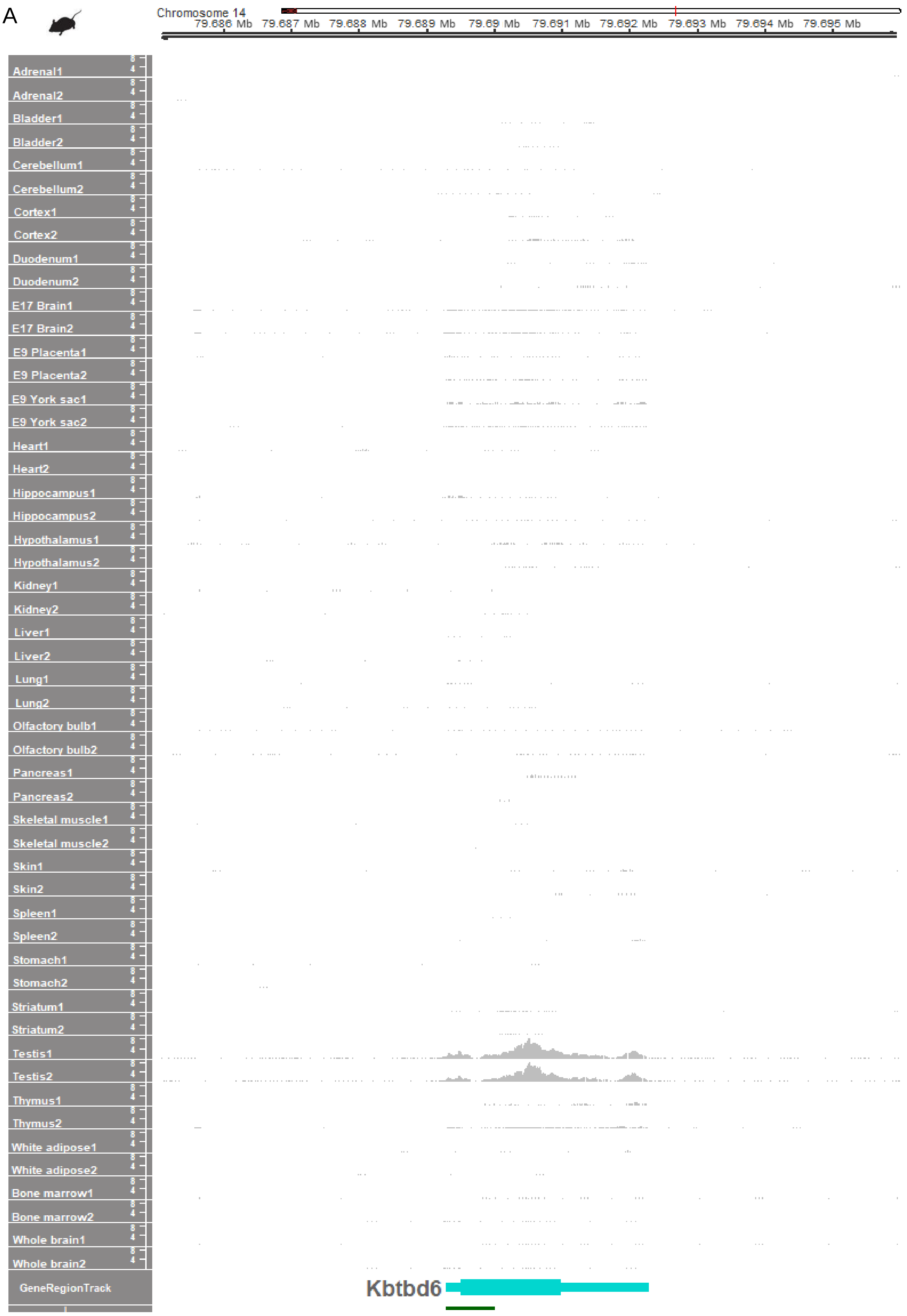

Chromosome 14
79.686 Mb
79.687 Mb
79.688 Mb
79.689 Mb
79.69 Mb
79.691 Mb
79.692 Mb
79.693 Mb
79.694 Mb
79.695 Mb
Adrenal1
Adrenal2
Bladder1
Bladder2
Cerebellum1
Cerebellum2
Cortex1
Cortex2
Duodenum1
Duodenum2
E17 Brain1
E17 Brain2
E9 Placenta1
E9 Placenta2
E9 York sac1
E9 York sac2
Heart1
Heart2
Hippocampus1
Hippocampus2
Hypothalamus1
Hypothalamus2
Kidney1
Kidney2
Liver1
Liver2
Lung1
Lung2
Olfactory bulb1
Olfactory bulb2
Pancreas1
Pancreas2
Skeletal muscle1
Skeletal muscle2
Skin1
Skin2
Spleen1
Spleen2
Stomach1
Stomach2
Striatum1
Striatum2
Testis1
Testis2
Thymus1
Thymus2
White adipose1
White adipose2
Bone marrow1
Bone marrow2
Whole brain1
Whole brain2
GeneRegionTrack
Kbtbd6

B

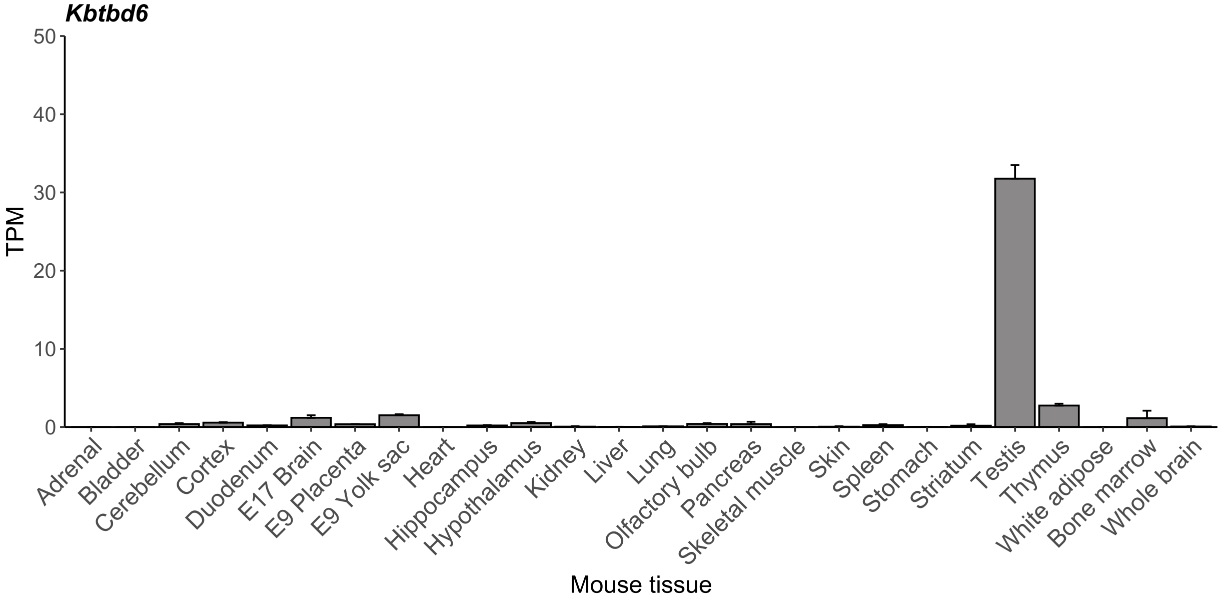

Supplementary Figure 9. Mouse *Kbtbd6* mRNA expression. (**A**) RNA-seq read coverages in various mouse tissues that were normalized to TPM using bamCoverage in deepTools. (**B**) Transcript quantification was performed on the same data in (A) using Salmon and TPM values are displayed indicating testis-enriched expression of mouse *Kbtbd6*. Values are presented as mean ± SEM for biological duplicates. RNA-seq raw data were derived from a dataset under accession number SRP020526. E, embryonic day. Testis-enriched expression of mouse *Kbtbd6* is also shown in bioGPS (http://biogps.org) based on microarray data (GSE10246).

A

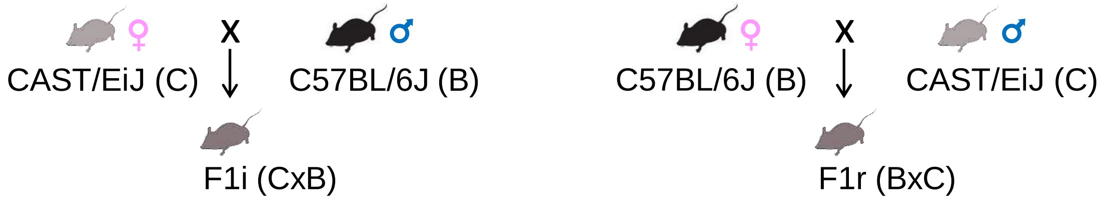

B

SNP ID	Map Position (GRCm39/ mm39)	Gene & Category	Variation Type	Allele Summary (all strains)	CAST/EiJ	C57BL/6J (ref)
rs32190201	chr14:79690553	Kbtbd6 Coding-Nonsynonymous	SNP	T/C	C	T
rs32190199	chr14:79690610	Kbtbd6 Coding-Nonsynonymous	SNP	T/C	C	T
rs240822699	chr14:79690801	Kbtbd6 Coding-Nonsynonymous	SNP	A/G	G	A
rs32190197	chr14:79690839	Kbtbd6 Coding-Synonymous	SNP	A/T	T	A

C

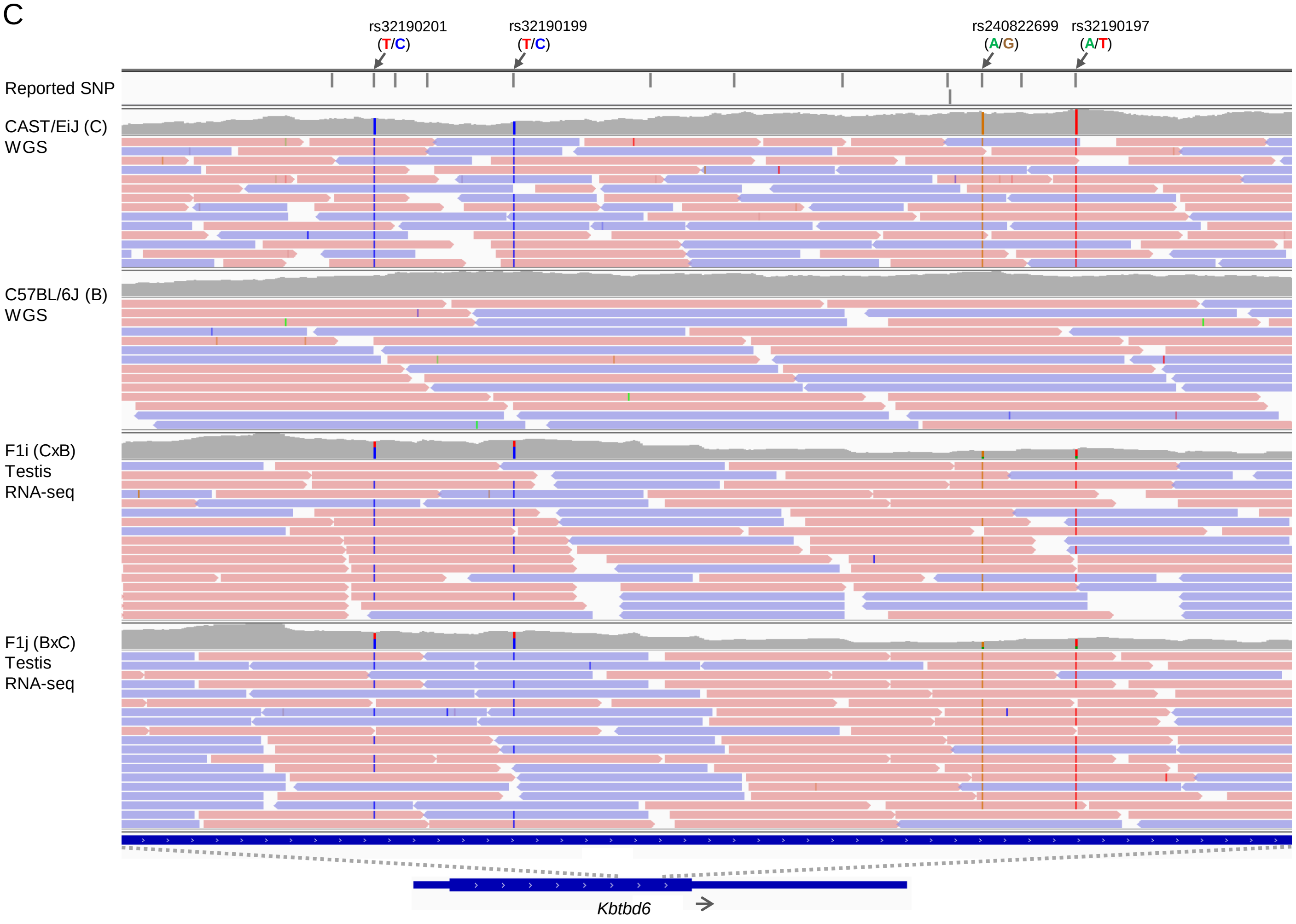

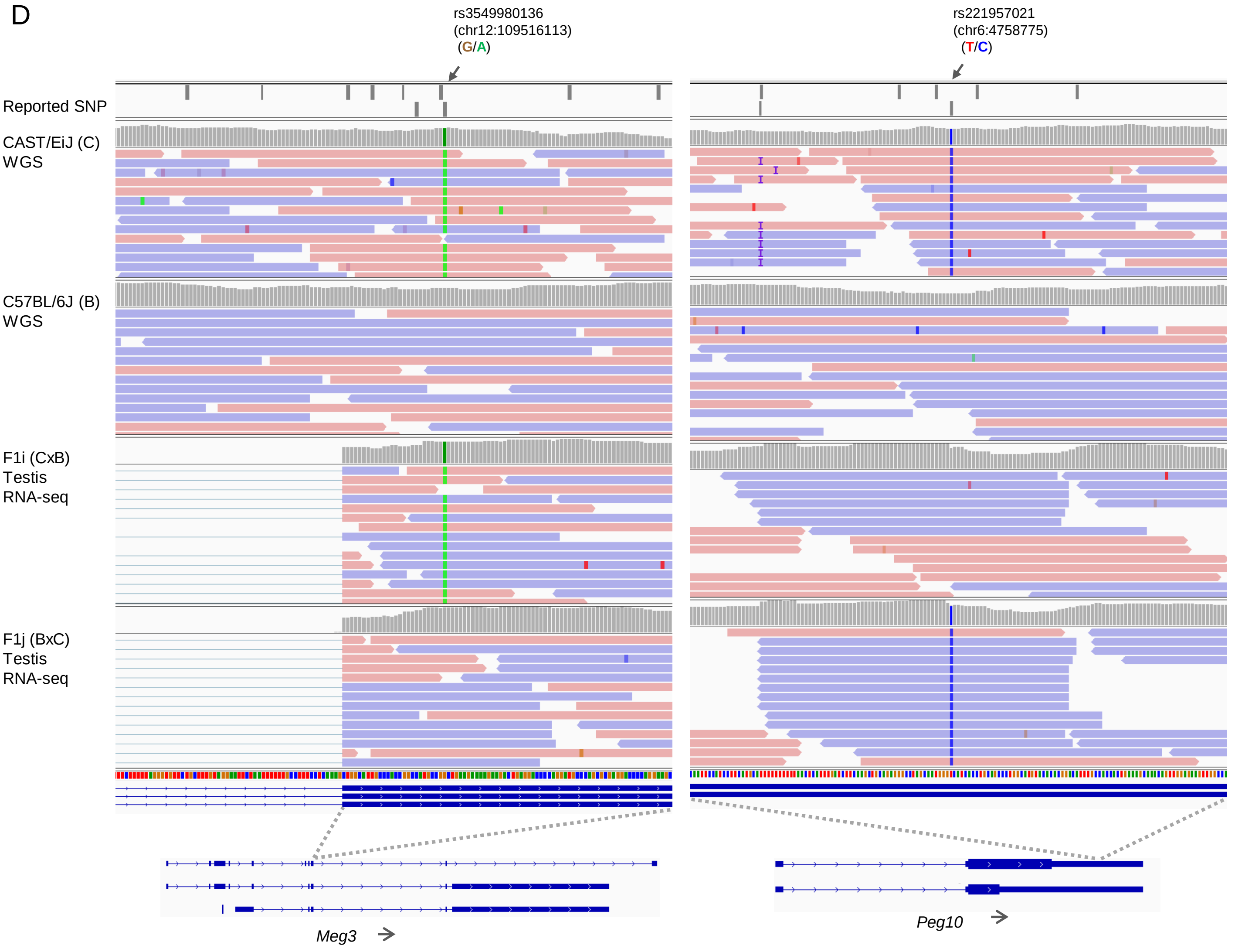

Supplementary Figure 10. Biallelic expression of mouse *Kbtbd6* in F1i and F1r. (**A**) F1 mice from initial cross (F1i) and reciprocal cross (F1r). (**B**) SNP information for mouse *Kbtbd6*. (**C**) Read coverages of mouse *Kbtbd6*. The raw WGS data of CAST/EiJ and C58BL/6J were retrieved from the NCBI/SRA under accession number ERP000042 (PRJEB1995) and SRX10207286 (PRJNA705216), respectively. The raw RNA-seq data of testis from F1i and F1r mice were retrieved from the NCBI/SRA under accession number SRP020526 (PRJNA196210). (**D**) Maternally expressed 3 (*Meg3*) and paternally expressed 10 (*Peg10*) are displayed as controls for maternally and paternally expressed imprinted genes, respectively, with SNPs originated from either the female (left) or male (right) CAST/EiJ mice .

A

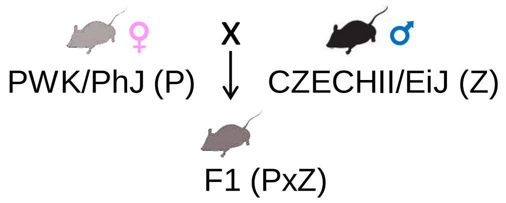

B

SNP ID	Map Position (GRCm39/ mm39)	Gene & Category	Variation Type	Allele Summary (all strains)	CZECHII/EiJ	PWK/PhJ
rs32190203	chr14:79690271	Kbtbd6 Coding-Nonsynonymous	SNP	G/C	C	G
rs32190199	chr14:79690610	Kbtbd6 Coding-Nonsynonymous	SNP	T/C	C	T
rs240822699	chr14:79690801	Kbtbd6 Coding-Nonsynonymous	SNP	A/G	G	A

C

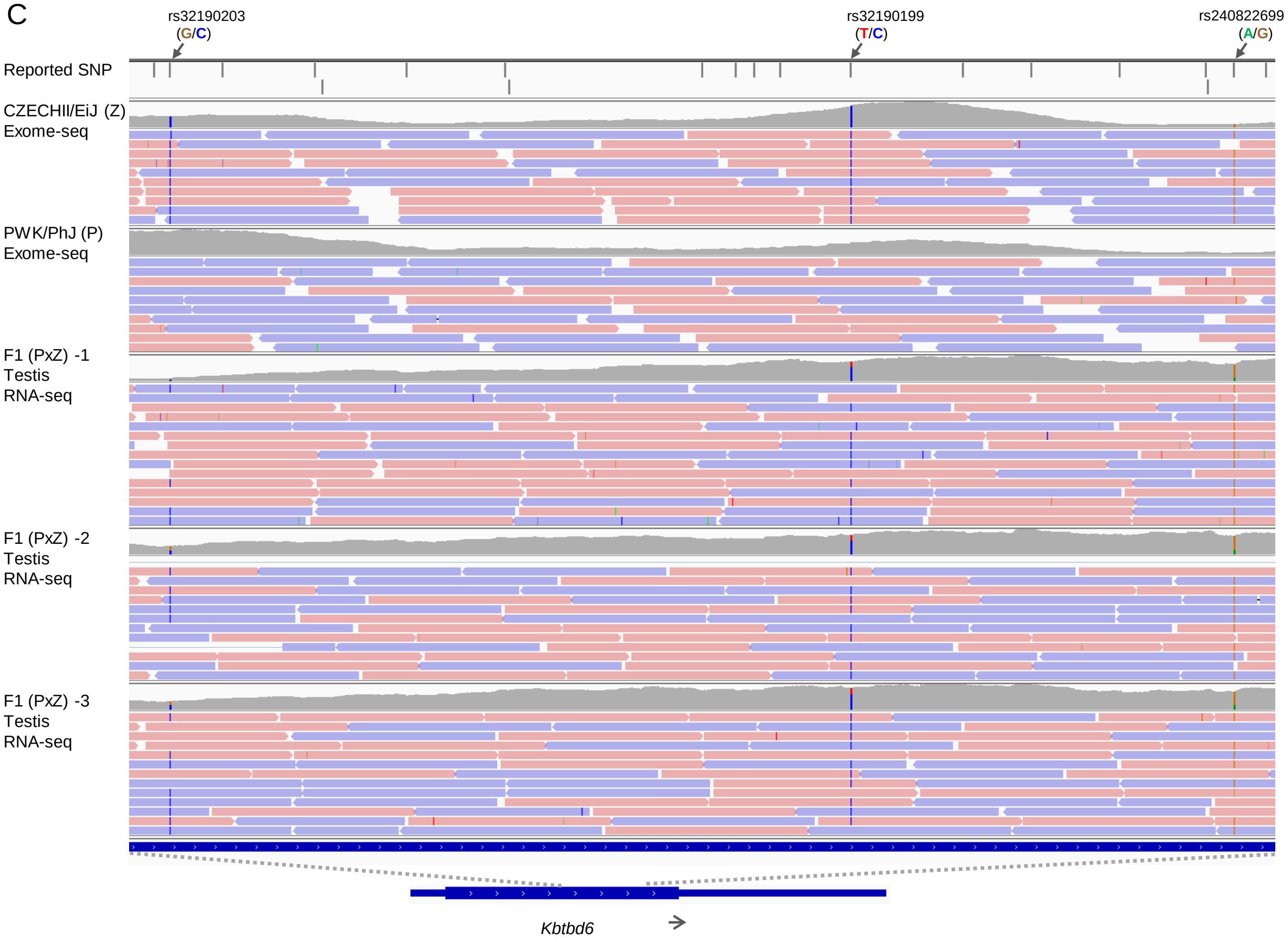

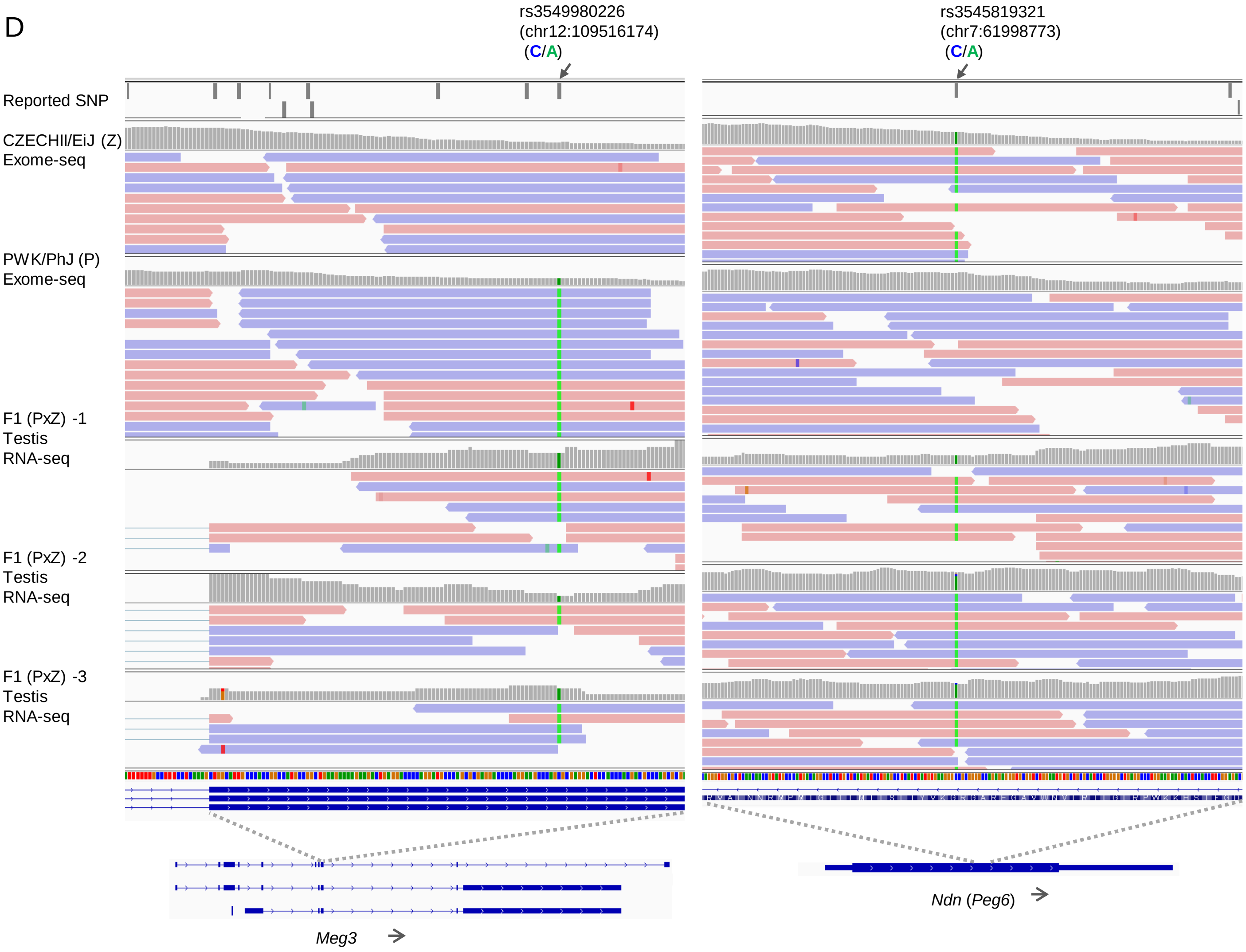

Supplementary Figure 11. Biallelic expression of mouse *Kbtbd6* in F1. (**A**) F1 mice from cross. (**B**) SNP information for mouse *Kbtbd6*. (**C**) Read coverages of mouse *Kbtbd6*. The raw exome-seq data of CZECHII/EiJ and PWK/PhJ were retrieved from the NCBI/SRA under accession number SRP075865 (PRJNA323493). The raw RNA-seq data of testis from F1 mice were retrieved from the NCBI/SRA under accession number SRP059387 (PRJNA286765). (**D**) *Meg3* and necdin (*Ndn*, aka. *Peg6*) are displayed as controls for maternally and paternally expressed imprinted genes, respectively, with SNPs originated from either the PWK/PhJ (female) or CAST/EiJ (male) mice.

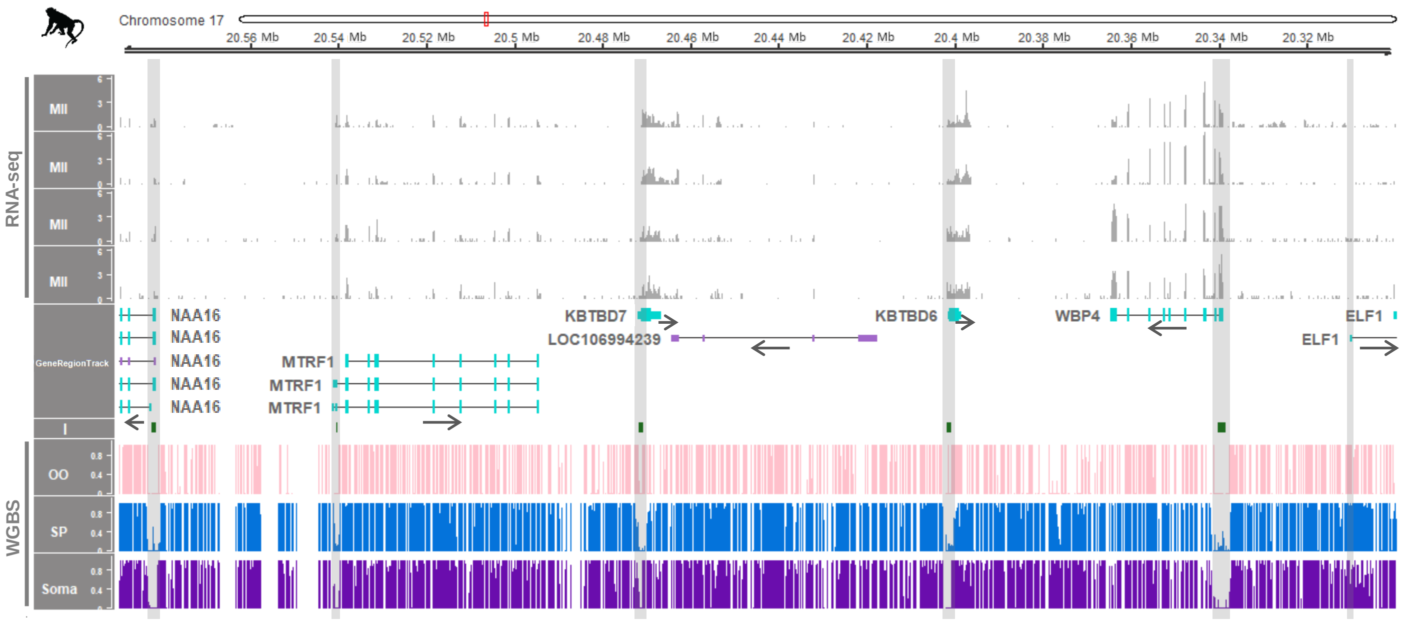

Supplementary Figure 12. Non-transcriptional initiation and unmethylation at the CpG promoter of *KBTBD6* in the rhesus monkey. Expressed transcripts from the *MTRF1-WBP4* interval in rhesus monkey oocytes are displayed with read coverages normalized to TPM values. Annotated protein-coding and noncoding transcripts from the rheMac8 genome are color-coded in cyan and purple, respectively. Arrows denote transcriptional direction. Methylation ratios from WGBS are plotted with histogram lines. MII, MII stage oocytes; OO, oocytes; SP, sperm; Soma, somatic tissue (brain). Data were derived from GSE112536 (RNA-seq), GSE60166 (WGBS of oocytes and sperm), and GSE77124 (WGBS of brain).

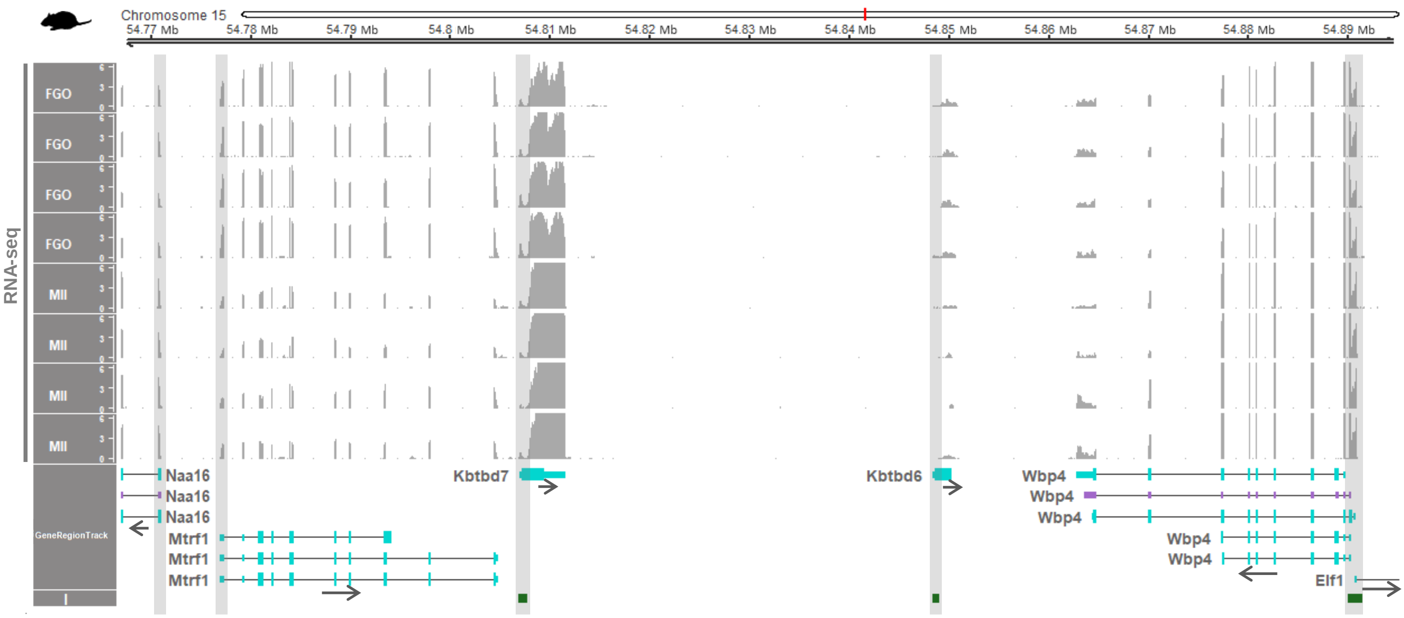

Supplementary Figure 13. Absence of transcriptional initiation at the CpG promoter of the *Kbtbd6* gene in the rat. The rat rn7 reference genome was used to map RNA-seq reads. Raw RNA-seq data from rat oocytes (GSE163620) were retrieved from NCBI GEO repository. FGO, fully grown oocytes; MII, MII stage oocytes.
